# (*Z*)-Selective Takai olefination of salicylaldehydes

**DOI:** 10.3762/bjoc.13.35

**Published:** 2017-02-20

**Authors:** Stephen M Geddis, Caroline E Hagerman, Warren R J D Galloway, Hannah F Sore, Jonathan M Goodman, David R Spring

**Affiliations:** 1 Department of Chemistry, University of Cambridge, Lensfield Rd, Cambridge, CB2 1EW, UK

**Keywords:** alkenyl iodides, salicylaldehydes, stereoselectivity, Takai olefination, transition state

## Abstract

The Takai olefination (or Takai reaction) is a method for the conversion of aldehydes to vinyl iodides, and has seen widespread implementation in organic synthesis. The reaction is usually noted for its high (*E*)-selectivity; however, herein we report the highly (*Z*)-selective Takai olefination of salicylaldehyde derivatives. Systematic screening of related substrates led to the identification of key factors responsible for this surprising inversion of selectivity, and enabled the development of a modified mechanistic model to rationalise these observations.

## Introduction

**Scheme 1:**
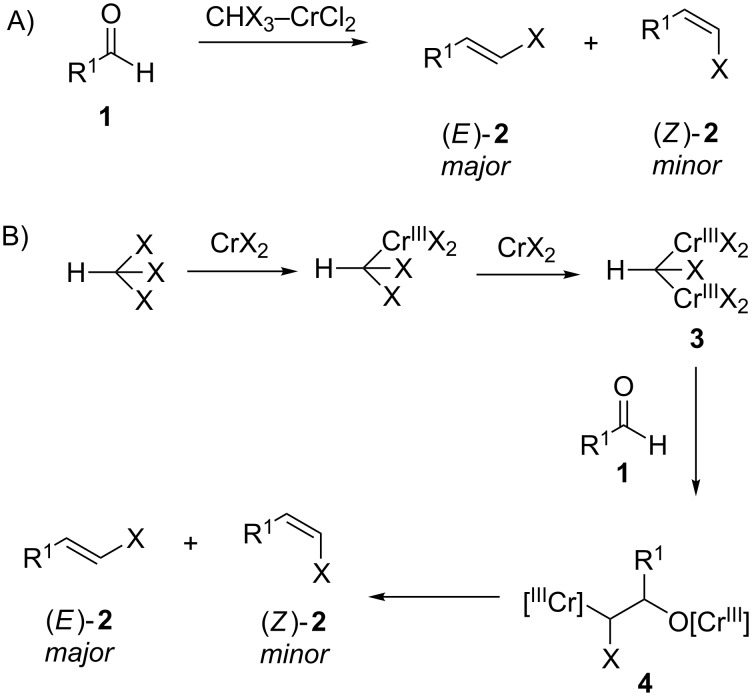
A) General overview of the Takai olefination for the formation of alkenyl halides **2** from aldehydes **1** and haloforms in the presence of Cr(II)Cl_2_. B) Proposed course of the Takai olefination. Possible ancillary ligands omitted for clarity. X = Cl, Br or I.

The Takai olefination (or Takai reaction) is a method for the conversion of aldehydes **1** into the corresponding alkenyl halides **2** using a haloform–chromium(II) chloride (CHX_3_–CrCl_2_) system (Scheme 1A) [1–2]. It is believed that the haloform is first converted to a nucleophilic *gem*-dichromium species **3** that then attacks the carbonyl group of the aldehyde to generate a β-oxychromium species **4** (Scheme 1B). Subsequent elimination leads to alkene formation. The Takai olefination can also be performed with geminal dihalide reagents rather than haloforms. In addition, the reaction can also be used to generate vinyl stannanes [3], silanes [4] and boronates [5].

One of the most significant features of the Takai olefination is that it is generally highly selective for the formation of (*E*)-alkenes ((*E*):(*Z*)-product ratios are typically around 4:1 or greater). This selectivity has found wide use, particularly in the field of total synthesis where it is often used to install vinyl iodides with high levels of geometric purity which can then be utilised in metal-catalysed cross-coupling reactions [2,6].

**Scheme 2:**
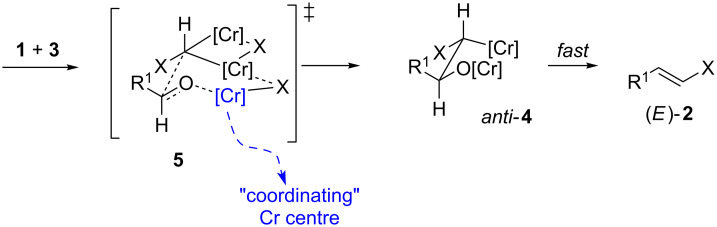
Proposed model for the chromium(II)-mediated homologation of aldehydes to form (*E*)-alkenes. Hodsgon et al. [7] and Takai et al. [5] have hypothesised that the addition of the *gem*-dichromium species **3** to aldehyde **1** proceeds via a six-membered pseudo-chair transition state **5**. X = Cl, Br or I. Other ligands on chromium omitted for clarity*.*

Hodgson et al. [7] and later Takai et al. [5] have proposed very similar models to explain the (*E*)-stereoselectivity observed in the chromium(II)-mediated homologation of aldehydes to alkenes (including vinyl halides). The salient features of both models are the same (Scheme 2). It is presumed that the addition of the *gem*-dichromium species **3** to the aldehyde **1** proceeds via a six-membered pseudo-chair transition state **5** containing two chromium ions bridged by a halogen. The less sterically hindered equatorial positions are occupied by the aldehyde substituent (R^1^ in Scheme 2) of **1** and halide group (X) of **3** and the aldehyde oxygen is thought to be coordinated to one of the Cr centres (the “coordinating” Cr centre highlighted in Scheme 2). The resulting β-oxychromium species adduct **4** will exist in a conformation where the two hydrogen atoms across the single bond are *anti* to each other (referred to as the *anti*-**4** conformation, Scheme 2). *Syn*-elimination is then thought to take place before rotation of the formed bond to give the (*E*)-configured olefin, (*E*)-**2** [5]. The minor (*Z*)-configured product, (*Z*)-**2**, presumably results from the reaction through a less favourable transition state which is nearly identical to **5** but differs in that either the R^1^ substituent originating from the aldehyde or the X substituent originating from the *gem*-dichromium species is in the sterically more hindered axial position at the analogous position of the ring (Hodgson et al. suggest the X substituent will be axial and the R^1^ group remains equatorial in this less favourable transition state) [7].

**Scheme 3:**
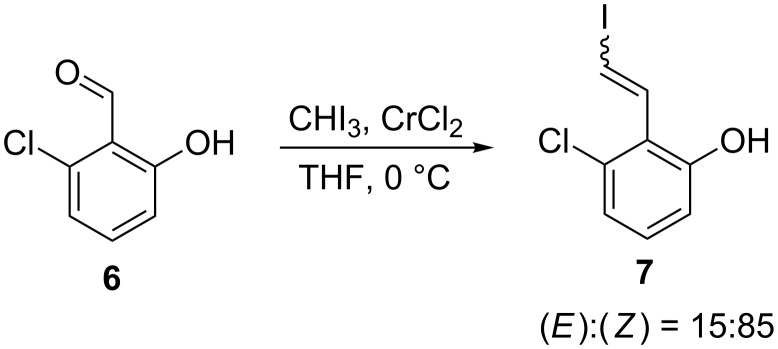
An unusually high level of (*Z*)-stereoselectivity was observed in the Takai olefination of **6**. (*E*):(*Z*)-ratio determined by ^1^H NMR analysis of crude material obtained after reaction work-up. See Supporting Information File 1 for more information.

As part of an on-going total synthesis programme, we subjected 6-chlorosalicylaldehyde (**6**) to standard Takai olefination conditions using iodoform (see Supporting Information File 1 for details) to form alkenyl iodide product **7** (Scheme 3). To our surprise a large excess of the (*Z*)-isomer of **7** relative to the corresponding (*E*)-isomer was observed in the crude material (approx. ratio of (*E*)-**7**:(*Z*)-**7** of 15:85 according to ^1^H NMR analysis, see Supporting Information File 1 for more information). There are several other examples of poor (*E*)-selectivity in the literature [8–10]; however, no detailed investigations as to the origins of this effect have been carried out. We therefore embarked on a study to understand the influence of the substrate structure upon the (*E*):(*Z*)-product ratio under Takai olefination conditions.

## Results and Discussion

In order to understand the influential functional groups responsible for the (*Z*)-selectivity of **6**, a number of *ortho*-substituted benzaldehydes were subjected to Takai olefination conditions; the results are summarised in Table 1 [11].

**Table 1:** (*E*):(*Z*) product ratios for Takai olefination of ortho-substituted benzaldehydes.

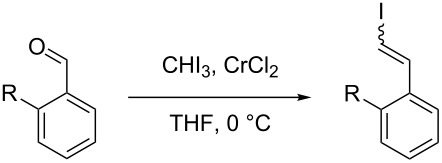				

Entry	R	Substrate	Product	(*E*):(*Z*) Ratio^a^

1	H	**8**	**9**	100:0
2	Me	**10**	**11**	78:22
3	OH	**12**	**13**	44:56
4	OAc	**14**	**15**	67:33
5	NH_2_	**16**	**17**	decomposition
6	NHAc	**18**	**19**	83:17
7	Cl	**20**	**21**	69:31

^a^Determined by analysis of ^1^H NMR spectra of crude materials obtained after reaction work-up. See Supporting Information File 1 for more information.

Unsubstituted benzaldehyde (Table 1, entry 1) gave the usual high (*E*)-selectivity expected of the Takai olefination (Takai et al. reported an (*E*):(*Z*)-ratio of 94:6 under identical conditions). The introduction of an *ortho*-methyl group (Table 1, entry 2) resulted in a slight increase in the amount of (*Z*)-product; however, this effect was dwarfed by the introduction of an *ortho*-OH group (Table 1, entry 3), where the (*Z*)-alkene was produced in a higher ratio. This effect was attenuated upon acetyl protection of the OH group (Table 1, entry 4). Attempted Takai olefination of the *ortho*-NH_2_ substrate resulted only in decomposition of the starting materials (Table 1, entry 5), whereas the acetyl protected analogue gave largely (*E*)-selective results (Table 1, entry 6). The presence of an *ortho*-Cl resulted in a moderate amount of (*Z*)-product (Table 1, entry 7).

**Scheme 4:**
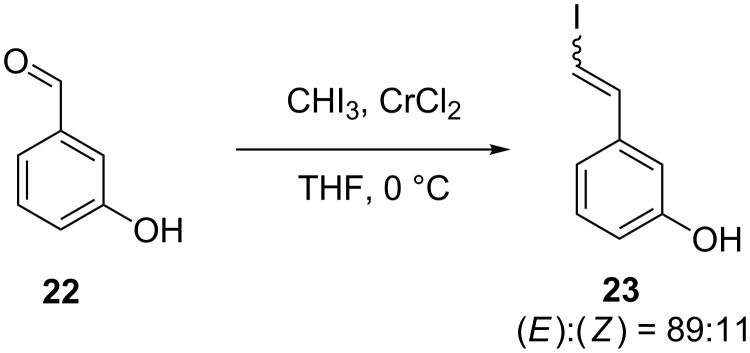
Takai olefination of *meta*-hydroxybenzaldehyde.

The above results imply that the presence of an OH group favours the generation of the (*Z*)-product. The product ratio was then determined for *meta*-OH benzaldehyde **22**; however, this resulted in a much lower amount of (*Z*)-product (Scheme 4), implying that an *ortho*-relationship is optimum for the (*Z*)-selectivity. Interestingly, comparison of our initial results of the Takai olefination of **6** with entries 3 and 7 in Table 1, we can conclude that the presence of a Cl substituent in addition to the *ortho*-OH results in a higher (*Z*)-selectivity than for either substituent in isolation.

In order to further investigate this cooperative effect, the (*E*):(*Z*)-product ratios were determined for various substituted salicylaldehydes (with *ortho*-OH substitution already demonstrated as optimum for favouring the (*Z*)-product); the results are shown in Table 2 along with the previously obtained result for **6**.

**Table 2:** (*E*):(*Z*)-product ratios for Takai olefination of substituted salicylaldehydes. *Ortho*- and *meta*-nomenclature refers to the substituents’ relationship to the aldehyde moiety.

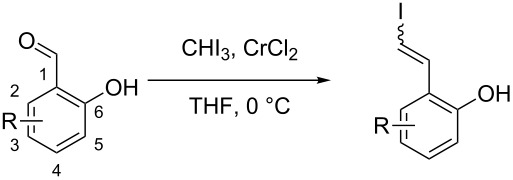							

Entry	R	2-Substituted (*ortho*)			3-Substituted (*meta*)		
		Substrate	Product	(*E*):(*Z*) Ratio^a^	Substrate	Product	(*E*):(*Z*) Ratio^a^

1	F	**24**	**25**	26:74	**30**	**31**	36:64
2	Cl	**6**	**7**	15:85	**32**	**33**	25:75
3	Br	**26**	**27**	13:87	**34**	**35**	23:77
4	I	**28**	**29**	26:74	**36**	**37**	32:68
5	OMe	–	–	–	**38**	**39**	72:28
6	CO_2_Me	–	–	–	**40**	**41**	36:64

^a^Determined by analysis of ^1^H NMR spectra of crude materials obtained after reaction work-up. See Supporting Information File 1 for more information.

**Scheme 5:**
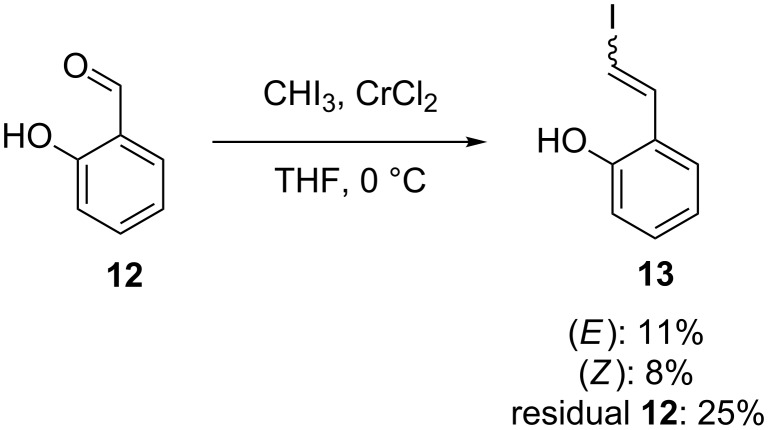
Yield for both products and residual starting material following a scaled up Takai olefination of salicylaldehyde (**12**) using optimised work-up conditions. (Yields determined from crude NMR using 1,3,5-trimethoxybenzene as an internal standard. See Supporting Information File 1 for details).

In order to determine whether the (*E*):(*Z*)-ratios presented above reflect those which might be obtained during the course of a synthetic scheme, the crude yield was determined (using an internal NMR standard) for a larger scale reaction. Unsubstituted salicylaldehyde (**12**) was chosen for this experiment, as it contains the core structure present in all the examples above, and gives approximately equal amounts of the (*E*)- and (*Z*)-products (Scheme 5). Initially, low recovery of material was observed, which we postulate was due to coordination (vide supra) of both starting material and product to the superstoichiometric amount of chromium present. However, work-up conditions were found (see Supporting Information File 1 for details) which facilitated the recovery of material accounting for 44% of the material submitted to the reaction conditions. Volatility of the starting material may also account for the moderate total yield. Critically, the ratio of (*E*):(*Z*)-products did not show significant deviation from that determined during the smaller scale tests, and sufficient material was recovered to give confidence that these ratios reflect the dominant behaviour of the reaction.

**Figure 1:**
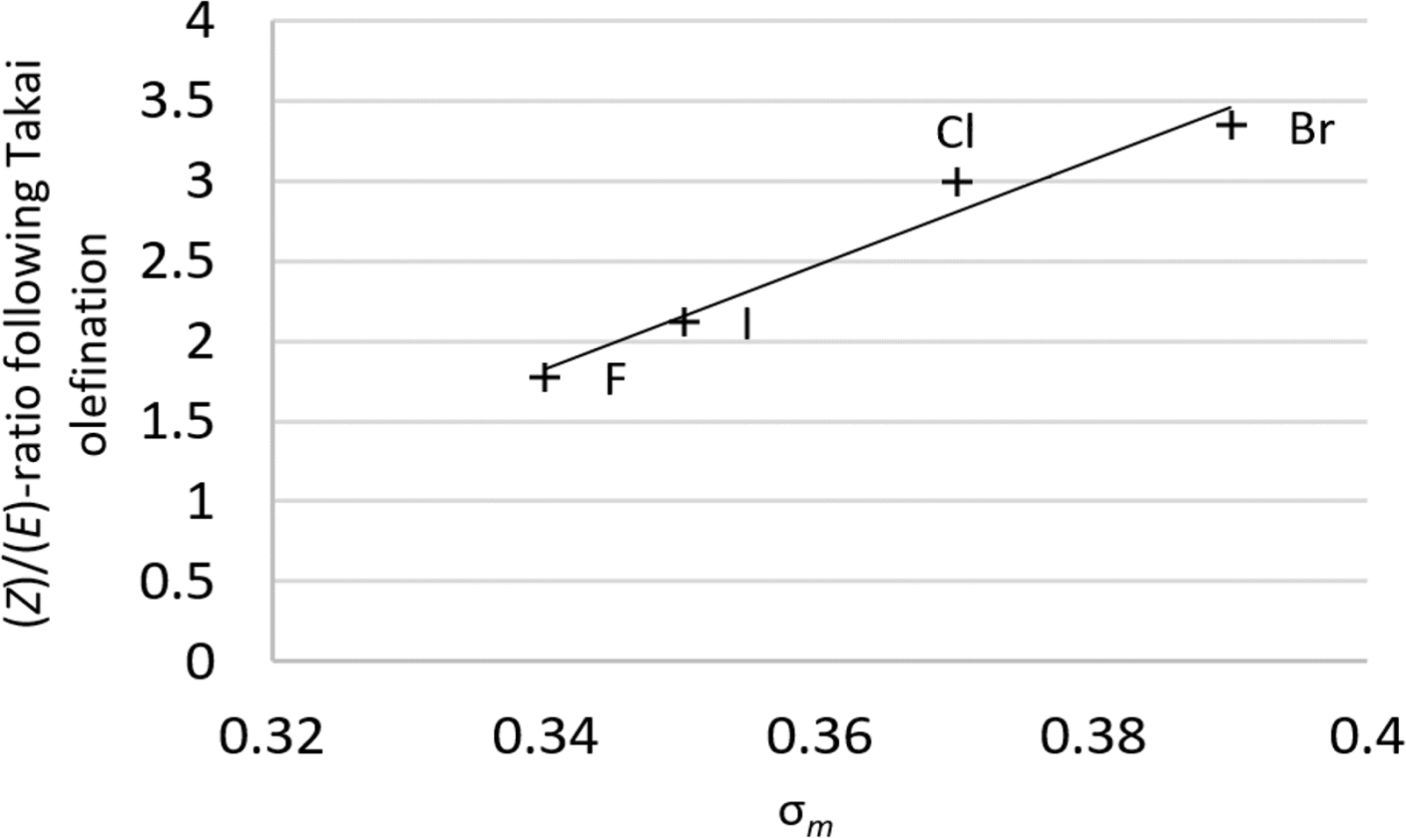
Positive correlation between the amount (*Z*)-product and σ*_m_* for the series of *meta*-halogenated salicylaldehydes.

Using the appropriate Hammett substituent constant (σ*_m_*) [12] as a measure of the net electron-withdrawing effect of a given substituent, for the *meta*-halogenated series, a positive correlation with the relative amount of (*Z*)-product was observed (Figure 1). Whilst Hammett parameters are not tabulated for *ortho*-substitution, precluding an analogous quantitative analysis, the trend for *ortho*-halogenated substrates is qualitatively similar, although with even higher amounts of (*Z*)-product. This implies that electron-withdrawing groups favour the production of the (*Z*)-product upon Takai olefination, with the effect dropping off with increasing distance of the electron-withdrawing group from the aldehyde and hydroxy moieties. To further test this theory, *meta*-OMe and CO_2_Me substrates were subjected to the reaction conditions (Table 2, entries 5 and 6). As expected, the electron-donating methoxy group caused a drop in the amount of (*Z*)-product, and conversely for the electron-withdrawing CO_2_Me group.

Our results highlight two key points: a) generation of the (*Z*)-product during Takai olefination of benzaldehydes is favoured by the presence of an *ortho*-OH group; b) this effect is magnified if the substrate also possesses electron-withdrawing groups. These observations should prove useful during the planning of synthetic schemes by providing warning that a planned Takai olefination may not proceed with the expected (*E*)-selectivity. Additionally, with careful design of substrates, synthetic routes utilising (*Z*)-selective Takai olefinations can now be considered, a strategy which has been hitherto unprecedented.

**Scheme 6:**
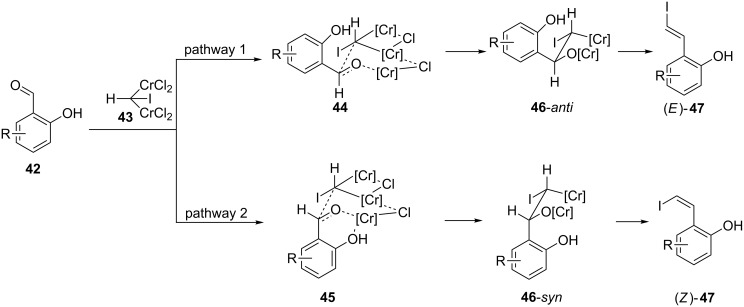
Proposed mechanism for (*Z*)-selective Takai olefination, whereby coordination of the *ortho*-OH to the neighbouring Cr centre causes the aldehyde substituent to adopt a pseudo-axial orientation.

A control experiment showed that, in the presence of 0.5 equivalents of salicylaldehyde, benzaldehyde was converted to the expected (*E*)-product (See Supporting Information File 1). This precludes the possibility that the salicylaldehydes are functioning as ligands for chromium. In light of this and the above observations, we propose an alternative pathway by which the reaction may proceed in order to favour the (*Z*)-product (Scheme 6). Our proposed mechanism commences with nucleophilic addition of *gem*-dichromium species to the aldehyde moiety via a six membered pseudo-chair transition state, as is generally accepted [5,7]. However, rather than proceeding via the transition state which places most groups in a pseudo-equatorial position (pathway 1), we propose that for the substrates under discussion, the *ortho*-OH group is able to coordinate to the neighbouring Cr centre if the aldehyde aromatic substituent occupies a pseudo-axial conformation, thus favouring pathway 2. Following *syn*-elimination, this then leads to the observed (*Z*)-olefin products, (*Z*)-**47**. Whilst further experimental data is required in order to bolster this hypothesis, our proposed mechanism should act as a starting point for future detailed mechanistic investigations.

Our observation that this alternative pathway is favoured by electron-withdrawing substituents initially seems counter-intuitive, as it might be predicted that these could attenuate the *ortho*-hydroxy’s ability to coordinate to the Cr centre. Although the effect appears to correlate with lowered p*K_a_* of this hydroxy group, moderate amounts of (*Z*)-product are generated when this group is acetylated (**14**) and the relevant proton is therefore absent. This implies that increased acidity is not the primary factor at play. Instead, based on the higher (*Z*)-selectivity observed whenever the electron-withdrawing group was closer to the aldehyde moiety (Table 2), we propose that it is withdrawal of electron density from this group which is the key. An electron-deficient aldehyde is less able to supply electron density to the Cr centre in transition state **44**. In order to counteract this deficit in electron donation, the *ortho*-OH chelates the Cr, thus favouring the pseudo-axial conformation, as discussed above.

## Conclusion

Following our observation of unusually high levels of (*Z*)-olefin product following Takai olefination of a particular aromatic substrate, systematic screening of related substrates revealed the characteristics a substrate should possess in order to lead to (*Z*)-selective Takai olefination. The substrate should possess an *ortho*-OH group, and the effect will be larger if the substrate also possesses electron-withdrawing substituents. These observations will allow synthetic chemists to predict a priori whether a planned Takai olefination will proceed with lower (*E*)-selectivity than predicted, and could also lead to synthetic routes based upon the (*Z*)-selective Takai olefination of suitably designed substrates. In addition, a modified mechanistic model was proposed accounting for these observations.

## Supporting Information

File 1 Experimental procedures and analytical data.
