# Supplementary material for: (Z)-Selective Takai olefination of salicylaldehydes
Source: Beilstein J Org Chem. 2017 Feb 20;13:323–8. doi: 10.3762/bjoc.13.35 (PMC5331342; doi:10.3762/bjoc.13.35)

Supporting Information File 1

for

(*Z*)-Selective Takai olefination of salicylaldehydes

Stephen M. Geddis‡, Caroline E. Hagerman‡, Warren R. J. D. Galloway, Hannah F. Sore, Jonathan M. Goodman and David R. Spring*

Address: Department of Chemistry, University of Cambridge, Lensfield Rd, Cambridge, CB2 1EW, UK

Email: David R. Spring - spring@ch.cam.ac.uk

* Corresponding author

‡equal contributors

Experimental procedures and analytical data

General experimental details

For reactions conducted under anhydrous conditions, glassware was dried overnight in an oven at 80 °C. All reactions, unless otherwise stated, were carried out under an atmosphere of nitrogen. All reactions were carried out at ambient (room) temperature unless otherwise stated.

Reagents were obtained from commercial sources and used without further purification. Standard practices were followed when handling air- or moisture-sensitive reagents.

Solvents were distilled prior to use. Solvents for anhydrous reactions were dried as follows: THF was dried over Na wire and distilled from a mixture of CaH_2 and $LiAlH_4$ with triphenylmethane as indicator. Diethyl ether was distilled from a mixture of CaH_2 and $LiAlH_4$. Dichloromethane, methanol, *n*-hexane, acetonitrile and toluene were distilled from CaH_2. Petroleum ether was distilled before use and refers to the fraction between 40 and 60 °C.

Yields refer to chromatographically and spectroscopically pure compounds unless otherwise stated. When possible, reactions were monitored by thin layer chromatography (TLC) performed on commercially-prepared glass plates pre-coated with Merck silica gel 60 F254. Visualisation was by quenching of ultraviolet fluorescence (v_{max} = 254 nm) or by staining with potassium permanganate. Retention factors (R_f) are quoted to 0.01.

LC–MS chromatographs were recorded on an HP/Agilent MSD LC-MS APCI 120-1000 full gradient ACq T = 1 min 1 µL.

Flash column chromatography was carried out using slurry-packed Merck 9385 Keiselgel 60 SiO_2 (230-400 mesh) under a positive pressure of compressed air.

Infrared spectra were recorded neat on a Perkin-Elmer Spectrum One spectrometer with internal referencing. Selected absorption maxima (v_{max}) are reported in wavenumbers (cm^{-1}) using the following abbreviations: w = weak; m = medium; s = strong; br = broad.

Proton magnetic resonance spectra were recorded using an internal deuterium lock at ambient probe temperatures (unless otherwise stated) on the following instruments: Bruker DRX-400 (400 MHz), Bruker Avance 400 QNP (400 MHz),

Bruker Avance 500 Cryo Ultrashield (500 MHz), and Bruker BB ATM 500 (500 MHz). Chemical shifts (δH) are quoted in parts per million (ppm), to the nearest 0.01 ppm, and are referenced to the residual non-deuterated solvent peak. Coupling constants (*J*) are reported in Hertz (Hz) to the nearest 0.1 Hz. Data are reported as follows: chemical shift, multiplicity (br = broad; s = singlet; d = doublet; t = triplet; q = quartet; quint = quintet; sept = septet; m = multiplet; or as a combination of these), coupling constant(s), integration, and assignment. The numbering/lettering on selected structures does not follow the IUPAC naming system and is used for the assignment of the ^{1}H NMR and ^{13}C NMR spectra. Proton assignments were determined either on the basis of unambiguous chemical shift, coupling pattern, by patterns observed in 2D experiments (^{1}H-^{1}H COSY, HMBC and HMQC) or by analogy to fully interpreted spectra for related compounds.

Carbon magnetic resonance spectra were recorded by broadband proton spin decoupling at ambient probe temperatures (unless otherwise stated) using an internal 191 deuterium lock on the following instruments: Bruker DRX-400 (100 MHz), Bruker Avance 400 QNP (100 MHz), Bruker Avance 500 Cryo Ultrashield (125 MHz), and Bruker BB ATM 500 (125 MHz). Chemical shifts (δC) are quoted in ppm, to the nearest 0.1 ppm, and are referenced to the deuterated solvent peak. Assignment was based on chemical shift, DEPT editing and where appropriate, HMQC and HMBC experiments or by analogy to fully interpreted spectra of related compounds.

General procedure for the Takai olefination

A 15 mL vial was charged with a stir bar and 4 Å molecular sieves and oven dried. It was then stoppered and flushed with argon. To this was added $CrCl_2$ (150 mg, 1.22

mmol, 6.0 equiv), which was weighed out under nitrogen. To this was added 4 mL of anhydrous THF and the suspension was cooled to 0 °C with stirring and bubbled through with argon. CHI_3 (161 mg, 0.41 mmol, 2.0 equiv) and benzaldehyde derivative (1.0 equiv) were dissolved in 2 mL of anhydrous THF and added to the THF/$CrCl_2$ suspension dropwise. The reaction was stirred for 3 hours under argon, as a deep green solution with suspended chromium. The reaction mixture was then quenched with 5 mL H_2O, and diluted with 25 mL Et_2O and 20 mL H_2O. The aqueous phase was extracted with 25 mL Et_2O and the combined organic layers were washed with 25 mL H_2O, dried ($MgSO_4$), and the solvent removed in vacuo.

Synthesis and characterisation

The following molecules are fully characterised, because they were either able to be purified or yielded pure *E* or *Z* isomer in the Takai olefination.

(*E*)-(2-Iodovinyl)benzene (9)

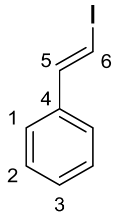

Benzaldehyde (265 µL, 2.60 mmol) was reacted according to the general procedure for the Takai olefination (vide supra). The product was purified by column chromatography (SiO_2, petroleum ether/Et_2O 40:1), followed by recrystallization from CH_2Cl_2 at 0 °C, followed by warming to RT to give the product as a yellow oil (310 mg, 1.34 mmol, 52%).

R_f = 0.86 (petroleum ether/EtOAc 10:1)

IR (neat) v_{max} = 3057 (w, C-H), 1595 (m, aromatic C=C);

^{1}H NMR (400 MHz, acetone-d6, 27°C) δ = 7.44 (d, J = 14.9 Hz, 1H; H-5 or H-6), 7.36-7.27 (m, 5H; H-1, H- 2, H-3), 6.83 (d, J = 14.9 Hz, 1H; H-5 or H-6);

^{13}C NMR (100 MHz, CDCl3, 27°C) δ = 145.5 (C-5), 138.1 (C-4), 129.2 (C-2), 128.8 (C-3), 126.5 (C-1), 77.1 (C-6).

These data are in accordance with those previously reported [1].

2-Formylphenyl acetate (14)

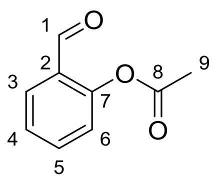

To a solution of salicylaldehyde (0.768 mL, 7.24 mmol, 1 equiv) in DMF (7 mL) at 0°C was added sodium hydride (174 mg, 7.24 mmol, 1 equiv) in small portions. The mixture was stirred at rt for 10 minutes, then acetic anhydride (747 mg, 7.31 mmol, 1.01 equiv) was added dropwise. After stirring overnight, the mixture was poured onto a mixture of iced water and 1 N aqueous HCl (20 mL). The aqueous layer was extracted with EtOAc (3 × 15 mL), then the combined organics were washed with $NaHCO_3$ (15 mL), water (15 mL), brine (15 mL) and dried ($MgSO_4$). The solvent was removed under reduced pressure, then the crude product was purified by flash chromatography using silica, eluting with 30% diethyl ether in hexane. Product was collected, along with what ^{1}H NMR revealed to be the dimethyliminium derivative of the product. This derivative was stirred overnight in a mixture of CH_2Cl_2 (25 ml) and 1 N HCl (25 mL), then the aqueous layer was extracted with EtOAc (3 × 15 mL) and the combined organics were washed with water (15 mL) then dried ($MgSO_4$).

Removal of solvent under reduced pressure yielded more product, which was combined with that produced earlier to yield a pale yellow liquid (0.372 g, 2.27 mmol, 31%).

R_f = 0.92 (10:1 CH_2Cl_2, MeOH)

IR: v_{max} = 2918 (w, C-H) 1757 (s, C(=O)O) 1689 (s, Ar C=O), 1603 (s, C=C), 1481 (w, C=C) 1458 (w, C=C)

^{1}H NMR: (500 MHz, $CDCl_3$): δ = 10.13 (1H, s, H1), 7.90 (1H, dd, J = 7.6, 1.5 Hz, H3), 7.66 (1H, ddd, J = 8.3, 7.7, 1.9 Hz, H5), 7.43 (1H, td, J = 7.6, 0.9 Hz, H4), 7.21 (1H, dd, J = 8.0, 0.9 Hz, H6), 2.42 (3H, s, H9)

^{13}C NMR: (125 MHz, $CDCl_3$): δ = 188.8 (C1), 169.3 (C8), 151.5 (C7) 135.3 (C3/4/5/6), 131.3 (C3/4/5/6), 126.4 (C3/4/5/6), 123.5 (C3/4/5/6), 128.0 (C2), 20.9 (C9)

The data are consistent with those previously reported [2].

N-(2-Formylphenyl)acetamide (18)

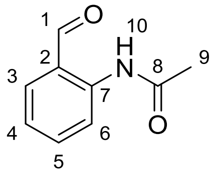

2-Aminobenzaldehyde (50.0 mg, 0.41 mmol, 1.0 equiv), acetic anhydride (51.1 mg, 0.50 mmol, 1.3 equiv) and triethylamine (67.2 mg, 0.62 mmol, 1.5 equiv) were dissolved in $CHCl_3$ (4 mL). The mixture was stirred at rt for 5 h, then refluxed at 60 °C for 12 h. Acetic anhydride (51.1 mg, 0.50 mmol, 1.3 equiv) and triethylamine (41.8 mg, 0.41 mmol, 1.0 equiv) were added, followed by reflux at 60 °C for 24 h. More

acetic anhydride (51.1 mg, 0.50 mmol, 1.3 equiv) and triethylamine (41.8 mg, 0.41 mmol, 1.0 equiv) were added, followed by reflux for a further 48 h. The mixture was then poured onto water (10mL), and the aqueous phase was extracted with $CHCl_3$ (3 × 5 mL). The combined organics were washed with 1 N aqueous HCl, water and brine, then dried ($MgSO_4$). Removal of the solvent under reduced pressure yielded the product as a white solid (24.5 mg, 0.15 mmol, 36%).

R_f = 0.90 (5:1 CH_2Cl_2, MeOH)

IR: v_{max} = 2974 (s, C-H), 1685 (w, C=O), 1584 (w, C=C), 1453 (m, C=C)

^{1}H NMR: (500 MHz, $CDCl_3$): δ = 11.16 (1H, s, H10), 9.95 (1H, s, H1), 8.76 (1H, d, J = 8.2 Hz, H3), 7.69 (1H, dd, J = 7.7, 1.6, H6), 7.64 (1H, ddd, J = 8.9, 7.3, 1.4 Hz, H4), 7.25 (1H, td, J = 7.5, 0.7 Hz, H5), 2.28 (3H, s, H9)

^{13}C NMR: (125 MHz, $CDCl_3$): δ = 195.6 (C1), 169.6 (C8), 141.0 (C7), 136.2, 136.0, 122.8, 121.4 (C2), 119.8, 25.4 (C9)

The data are consistent with those previously reported [3].

6-Bromosalicylaldehyde (26)

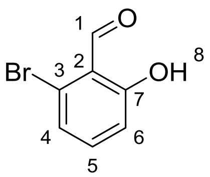

Sodium hydroxide (1.73 g, 44.2 mmol, 8.2 equiv) was dissolved in water (5 mL). 3-Bromophenol (0.96 g, 5.54 mmol, 1.0 equiv) was added and the mixture was heated to 75 °C. Chloroform (0.88 ml, 11.0 mmol, 2.0 equiv) was added and the resulting orange solution was stirred at 75 °C until reflux ceased (~45 m). After cooling to rt,

the mixture was poured onto 3N aqueous HCl (50 mL), followed by extraction with EtOAc (3 × 25 mL). The combined organics were dried ($MgSO_4$) then concentrated under reduced pressure. The resulting purple liquid was stirred with CH_2Cl_2 (10 mL) then filtered and concentrated. The crude product was purified by flash chromatography using silica, eluting with 10:1 petroleum ether 40–60, EtOAc, to yield the product as a white solid (72.0 mg, 0.358 mmol, 6%)

IR: v_{max} = 2902 (m, O-H), 1646 (s, C=O), 1610 (m, C=C), 1567 (m, C=C) 1438 (s, C=C))

^{1}H NMR: (400 MHz, $CDCl_3$): δ = 11.97 (1H, s, H8), 10.32 (1H, s, H1), 7.32 (1H, t, J = 8.2 Hz, H5), 7.15 (1H, dd, J = 7.8, 1.0 Hz, H4), 6.93 (1H, dt, J = 8.6, 1.0 Hz)

^{13}C NMR: (400 MHz, $CDCl_3$): δ = 197.5 (C1), 163.6 (C3), 137.1 (C7), 127.1, 124.1, 117.55, 117.45

The data are consistent with those previously reported [4].

6-Iodosalicylaldehyde (28)

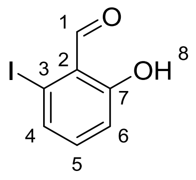

Sodium hydroxide (1.80 g, 46.0 mmol, 8.2 equiv) was dissolved in water (5 mL). 3-Iodophenol (1.23 g, 5.60 mmol, 1.0 equiv) was added and the mixture was heated to 75 °C. Chloroform (0.92 mL, 11.6 mmol, 2.1 equiv) was added and the resulting orange solution was stirred at 75 °C until reflux ceased (~45 m). After cooling to rt, the mixture was poured onto 3 N aqueous HCl (50 mL), followed by extraction with

EtOAc (3 × 25 mL). The combined organics were dried ($MgSO_4$) then concentrated under reduced pressure. The resulting thick purple liquid was stirred with CH_2Cl_2 (10 mL) then filtered and concentrated. The crude product was purified by flash chromatography using silica, eluting with 10:1 petroleum ether 40–60, EtOAc, to yield the product as a white solid (0.116 g, 0.469 mmol, 8%)

IR: v_{max} = 2973 (br m, O-H), 1643 (s, C=O), 1596 (m, C=C), 1561 (s, C=C), 1479 (w, C=C), 1433 (s, C=C)

^{1}H NMR: (500 MHz, $CDCl_3$): δ = 12.07 (1H, s, H8), 10.09 (1H, s, H1), 7.51 (1H, dd, J = 7.6, 0.9 Hz, H4), 7.15 (1H, t, J = 8.1 Hz, H5), 7.00 (1H, dt, J = 8.6, 0.9 Hz, H6)

^{13}C NMR: (125 MHz, $CDCl_3$): 202.4 (C1), 163.6, 137.7, 131.7, 119.0, 118.9, 101.9

Takai olefination NMR data

^{1}H NMR and COSY data were obtained for all Takai olefination products. From COSY it was possible to correlate the two peaks of the alkene for each olefin isomer, and therefore incontrovertibly assign the alkene peaks. The aromatic ring peaks of the substrates largely overlapped with each other, and were unimportant to determination of *E/Z* ratio. Reported below is ^{1}H NMR data for one or both of the alkene peaks for each isomer, depending on whether both were clearly separated and therefore able to be integrated. Integral values are reported relative to the most deshielded alkene peak, and the *E/Z* ratios thereby determined.

(*E*)-3-Chloro-2-(2-iodovinyl)phenol and (*Z*)-3-chloro-2-(2-iodovinyl)phenol (7)

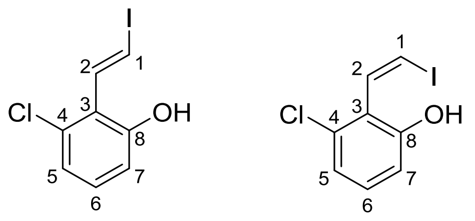

^{1}H NMR (400 MHz, acetone-d6, 27°C)

E isomer: *δ* = 7.77 (d, J = 14.8 Hz, 1.00 H, H-2), 7.51 (d, J = 14.7 Hz, 1.16 H, H-1)

Z isomer: *δ* = 7.32 (d, J = 8.0 Hz, 6.30 H, H-2), 7.02 (d, J = 8.0 Hz, 6.39 H, H-1)

E/Z ratio: 1.08/6.35, or 15:85.

(*E*)-1-(2-Iodovinyl)-2-methylbenzene and (*Z*)-1-(2-iodovinyl)-2-methylbenzene (11)

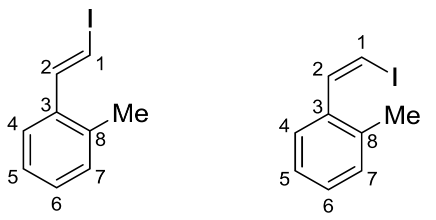

^{1}H NMR (400 MHz, acetone-d_6, 27 °C)

E isomer: *δ* = 7.73 (d, J = 14.8 Hz, 1.00 H; H-2), 6.97 (d, J = 14.7 Hz, 1.05 H; H-1).

Z isomer: *δ* = 6.67 (d, J = 8.4 Hz, 0.29 H; H-1), H-2 signal was overlapped, at ~7.45 ppm.

E/Z ratio: 1.03/0.29, or 78:22.

(*E*)-2-(2-Iodovinyl)phenol and (*Z*)-2-(2-iodovinyl)phenol (13)

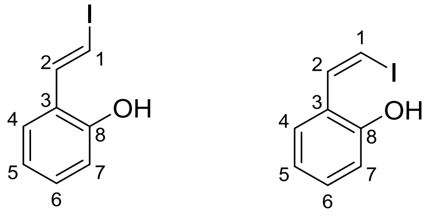

^{1}H NMR (400 MHz, acetone-d_6, 27 °C)

E isomer: δ = 7.71 (d, J = 14.9 Hz, 1.00 H; H-2), H-1 signal was overlapped, at ~7.1 ppm.

Z isomer: δ = 7.50 (d, J = 8.5 Hz, 1.20 H; H-2), 6.67 (d, J = 8.5 Hz, 1.17 H; H-1).

E/Z ratio: 1.00/1.18, or 44:56.

(*E*)-2-(2-iodovinyl)phenyl acetate and (*Z*)-2-(2-iodovinyl)phenyl acetate (15)

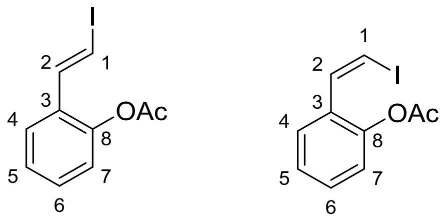

^{1}H NMR (400 MHz, acetone-d_6, 27 °C)

E isomer: δ = 7.54 (1.00H, d, J = 14.9 Hz, H-2), H2 signal was overlapped at ~7.20

Z isomer: δ = 6.84 (0.50H, d, J = 8.6 Hz, H-1), H-2 signal was overlapped at ~ 7.32 ppm

E/Z ratio: 1.00/0.50, or 67:33

(*E*)-*N*-(2-(2-iodovinyl)phenyl)acetamide and (*Z*)-*N*-(2-(2-iodovinyl)phenyl)acetamide (19)

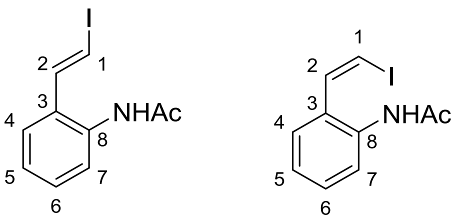

^{1}H NMR (400 MHz, acetone-d_6, 27 °C)

E isomer: δ = 7.04 (5.02 H, d, J = 14.7 Hz, H-1) H-2 signal was overlapped at ~7.69 ppm

Z isomer: δ = 7.42 (1.08 H, d, J = 8.2 Hz, H-2), 6.86 (1.02 H, d, J = 8.3 Hz, H-1)

E/Z ratio: 5.02/1.05, or 83:17

(*E*)-1-Chloro-2-(2-iodovinyl)benzene and (*E*)-1-chloro-2-(2-iodovinyl)benzene (21)

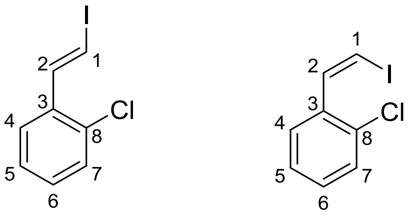

^{1}H NMR (400 MHz, acetone-d_6, 27 °C)

E isomer: δ = 7.81 (d, *J* = 14.9 Hz, 1.00 H, H-2), 7.28 (d, *J* = 14.9 Hz, H-8)

Z isomer: δ = 6.97 (d, *J* = 8.3 Hz, 0.50 H, H-1), H-2 signal was overlapped at ~7.45 ppm

E/Z ratio: 1.04/0.50, or 69:31

(*E*)-3-(2-Iodovinyl)phenol and (*Z*)-3-(2-iodovinyl)phenol (23)

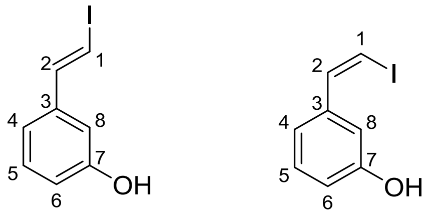

^{1}H NMR (400 MHz, acetone-d_6, 27 °C)

E isomer: δ = 7.44 (d, J = 14.9 Hz, 1.00 H; H-2), 7.06 (d, J = 14.9 Hz, 1.01 H; H-1).

Z isomer: δ = 7.37 (d, J = 8.6 Hz, 0.12 H; H-2), 6.68 (d, J = 8.6 Hz, 0.13 H; H-1).

E/Z ratio: 1.01/0.13, or 89:11.

(*E*)-3-Fluoro-2-(2-iodovinyl)phenol and (*Z*)-3-fluoro-2-(2-iodovinyl)phenol (25)

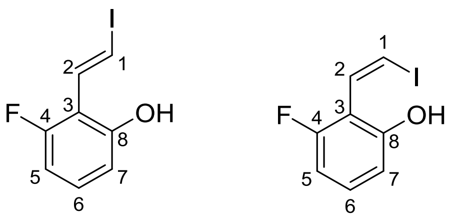

1H NMR (400 MHz, acetone-d_6, 27 °C)

E isomer: δ = 7.66 (d, J = 15.0 Hz, 1.00 H; H-2), H-1 signal was overlapped at ~7.35 ppm.

Z isomer: δ = 7.00 (d, J = 8.2 Hz, 2.91 H; H-1), H-2 signal was overlapped at ~7.30 ppm.

E/Z ratio: 1.00/2.91, or 26:74.

(E)-3-Bromo-2-(2-iodovinyl)phenol and (Z)-3-bromo-2-(2-iodovinyl)phenol (27)

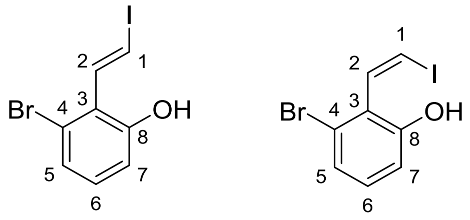

^{1}H NMR (400 MHz, acetone-d_6, 27 °C)

E isomer: δ = 7.73 (0.94H, d, J = 14.8 Hz, H-2), 7.48 (0.99H, d, J = 14.6 Hz, H-1)

Z isomer: δ = 7.29 (6.24H, d, J = 14.8 Hz, H-2), 7.00 (6.51H, d, J = 8.1 Hz, H-1)

E/Z ratio: 0.97/6.38, or 13:87

(E)-3-Iodo-2-(2-iodovinyl)phenol and (Z)-3-iodo-2-(2-iodovinyl)phenol (29)

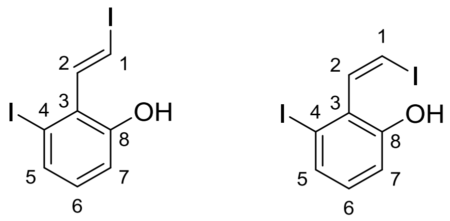

^{1}H NMR (400 MHz, acetone-d_6, 27 °C)

E isomer: δ = 7.61 (1.00H, d, J =14.4 Hz, H-2), H-1 peak overlapped at ~6.96 ppm

Z isomer: δ = 7.27 (2.91 H, d, J = 8.0 Hz, H-2), H-1 peak overlapped at ~6.96 ppm

E/Z ratio: 1.00/2.91, or 26:74

(*E*)-4-Fluoro-2-(2-iodovinyl)phenol and (*Z*)-4-fluoro-2-(2-iodovinyl)phenol (31)

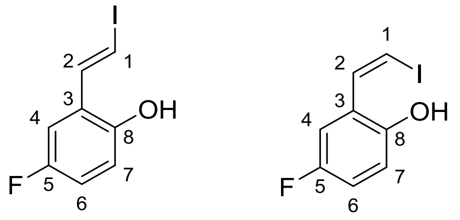

^{1}H NMR (400 MHz, acetone-d_6, 27 °C)

E isomer: *δ* = 7.68 (1.00H, d, J = 15.0 Hz, H-2), 7.22 (0.96 H, d, J = 15.0 Hz, H-1)

Z isomer: *δ* = 7.45 (1.71 H, d, J = 8.6 Hz, H-2), 6.75 (1.79 H, d, J = 8.6 Hz, H-1)

E/Z ratio: 0.98/1.75, or 36:64

(*E*)-4-Chloro-2-(2-iodovinyl)phenol and (*Z*)-4-chloro-2-(2-iodovinyl)phenol (33)

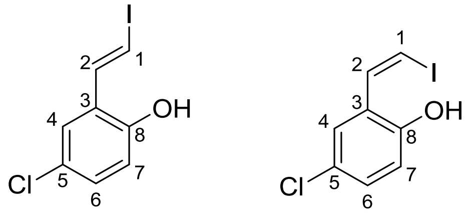

^{1}H NMR (400 MHz, acetone-d_6, 27 °C)

E isomer: *δ* = 7.67 (d, J = 15.0 Hz, 1.00 H; H-2), 7.27 (d, J = 15.0 Hz, 1.08 H; H-1).

Z isomer: *δ* = 6.80 (d, J = 8.5 Hz, 2.82 H; H-1), H-2 signal was overlapped at ~7.45 ppm.

E/Z ratio: 1.04/2.82, or 25:75.

(*E*)-4-Bromo-2-(2-iodovinyl)phenol and (*Z*)-4-bromo-2-(2-iodovinyl)phenol (35)

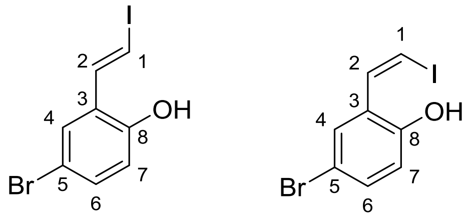

^{1}H NMR (400 MHz, acetone-d_6, 27 °C)

E isomer: δ = 7.64 (1.00 H, d, J = 15.0 Hz, H-2), peak for H-1 overlapped at ~7.26 ppm

Z isomer: δ = 7.40 (3.33 H, d, J = 9.8 Hz, H-2) 6.78 (3.34 H, d, J = 9.8 Hz, H-1)

E/Z isomer: 1.00/3.34, or 23:77

(*E*)-4-Iodo-2-(2-iodovinyl)phenol and (*Z*)-4-iodo-2-(2-iodovinyl)phenol (37)

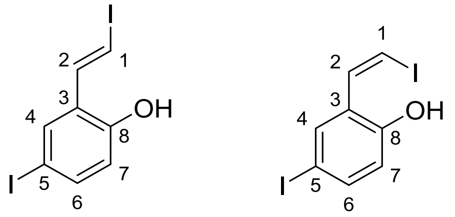

^{1}H NMR (400 MHz, acetone-d_6, 27 °C)

E isomer: δ = 7.63 (1.00 H, d, J = 14.6 Hz, H-2), 7.26 (0.87 H, d, J = 14.9 Hz, H-1)

Z isomer: δ = 7.40 (2.02 H, d, J = 8.3 Hz, H-2), peak for H-1 overlapped at ~6.78 ppm

E/Z ratio: 0.94/2.02, or 32:68

(*E*)-2-(2-Iodovinyl)-4-methoxyphenol and (*Z*)-2-(2-iodovinyl)-4-methoxyphenol (39)

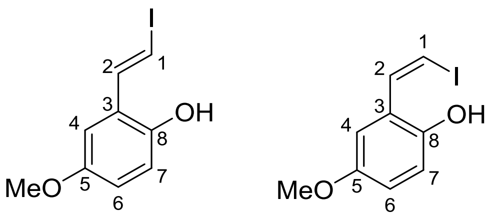

^{1}H NMR (400 MHz, acetone-d_6, 27 °C)

E isomer: δ = 7.70 (d, J = 14.9 Hz, 1.00 H; H-2), 7.15 (d, J = 14.8 Hz, 1.01 H; H-1).

Z isomer: δ = 7.50 (d, J = 8.5 Hz, 0.36 H; H-2), 6.68 (d, J = 8.4 Hz, 0.38 H; H-1).

E/Z ratio: 1.01/0.37, or 72:28.

(*E*)-Methyl 4-hydroxy-3-(2-iodovinyl)benzoate and (*Z*)-methyl 4-hydroxy-3-(2-iodovinyl)benzoate (41)

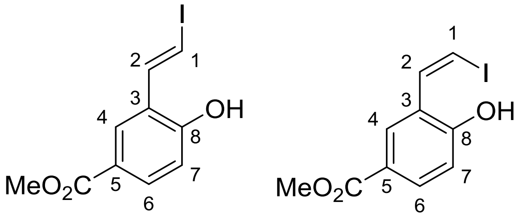

^{1}H NMR (400 MHz, acetone-d_6, 27 °C)

E isomer: δ = 7.28 (1.00H, d, J = 15.0 Hz, H-1), peak for H2 overlapped at ~7.71 ppm

Z isomer: δ = 7.47 (1.75 H, d, J = 8.6 Hz, H-2), peak for H1 was overlapped at ~6.80 ppm

E/Z ratio: 1.00/1.75, or 36:64

Scale-up experiment

A 50 mL round bottomed flask was charged with $CrCl_2$ (588 mg, 4.78 mmol) in a glove box. THF (16 mL) was added, cooled to 0 °C and bubbled through with argon with vigorous stirring. A solution of iodoform (628 mg, 1.60 mmol) and salicylaldehyde (118 mg, 0.97 mmol) was added dropwise at 0 °C to give a deep red mixture, which was stirred at 0 °C for 3 h 30 min. The reaction was quenched at room temperature by addition of poly(4-styrenesulfonic acid) solution (5 mL, Sigma-Aldrich, 18 wt % in H_2O), saturated aq $NaHCO_3$ (20 mL) and EtOAc (10 mL). The resulting suspension was stirred at room temperature for 1 h, then filtered through a pad of celite, washing the pad with EtOAc (30 mL). The aqueous phase was separated with EtOAc (2 × 25 mL), and the combined organics were washed with 1 M $Na_2S_2O_3$ (30 mL), brine (30 mL), dried ($MgSO_4$) and concentrated in vacuo to give 282 mg of crude material. This was dissolved in 10 mL of acetone to give a homogenous solution, 1 mL of which was concentrated in vacuo. 1,3,5-Trimethoxybenzene (4.8 mg, 0.029 mmol) was added, and the ^{1}H NMR spectrum determined (400 MHz, acetone-d_6, 27 °C). Relative integrals for each component were recorded as follows:

1,3,5-trimethoxybenzene: δ = 6.10 (s, 8.22H) (corresponds to 3H in the molecule)

Salicylaldehdye: δ = 7.62 (m, 2.28H)

E isomer: δ = 7.72 (d, J = 14. 7 Hz, 1.02H)

Z isomer: δ = 7.50 (d, J = 8.5 Hz, 0.76H)

Using these integrals, the amounts of each component in the bulk crude material were calculated as:

Salicylaldehdye: 0.24 mmol (25%)

E isomer: 0.11 mmol (11%)

Z isomer: 0.082 mmol (8%)

Control experiment

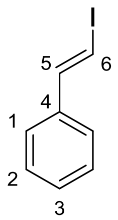

Benzaldehyde (1.0 equiv) and salicylaldehyde (0.5 equiv) were reacted according to general procedure for the Takai olefination using 9 equiv $CrCl_2$ and 3 equiv CHI_3. Crude NMR showed generation of only the *E* isomeric product from benzaldehyde; the corresponding *Z* isomer was undetectable.

^{1}H NMR (400 MHz, acetone-d6): δ = 7.51 (1 H, d, J = 15.0 Hz, H-5), 7.46-7.43 (2H, m, H-1), 7.38-7.29 (3H, m, H-2 & H-3)

References

[1] Bull, J. A.; Mousseau, J. J.; Charette, A. B. *Org. Synth*., **2010**, *87*, 170-177

[2] Pelletier, G.; Bechara, W. S.; Charette, A. B. *J. Am. Chem. Soc.*, **2010**, *132*, 12812-12819

[3] Ahmad, K.; Thomas, N. F.; Makhtar, M. R.; Noorbatcha, I.; Weber, J. F.; Nafiah, M. A.; Velu, S. S.; Takega, K.; Morita, H.; Lim, C.; Hadi, A. H. A.; Awang, K. *Tetrahedron*, **2009**, *65*, 1504-1516

[4] Rawat, M.; Prutyanov, V.; Wulff, W.D *J. Am. Chem. Soc.*, **2006**, *128*, 11044-11053

NMR Data

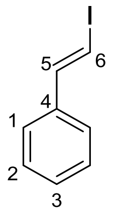
I
5
6
4
1
2
3

9

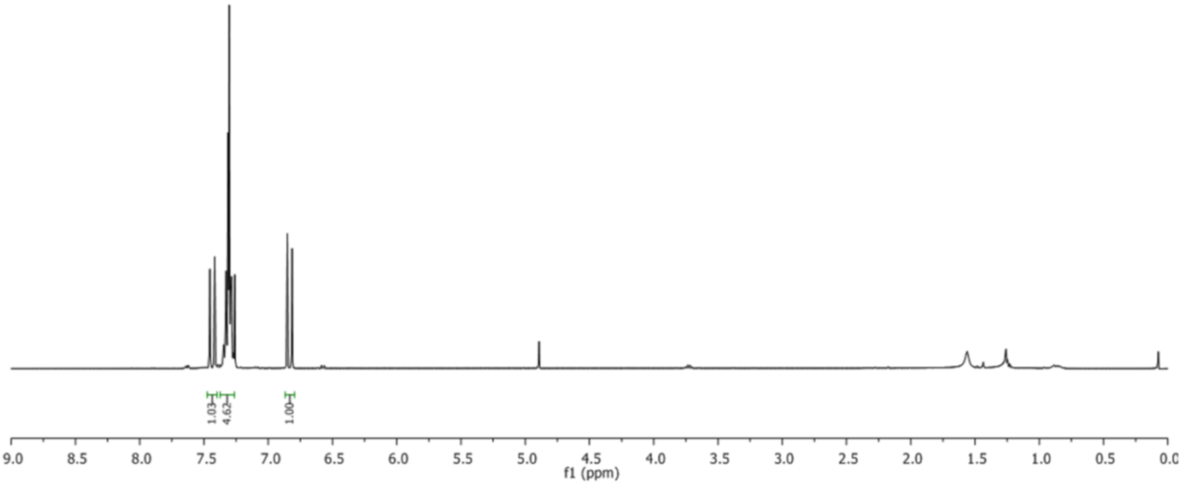
1.03
4.62
1.00
9.0 8.5 8.0 7.5 7.0 6.5 6.0 5.5 5.0 4.5 4.0 3.5 3.0 2.5 2.0 1.5 1.0 0.5 0.0
f1 (ppm)

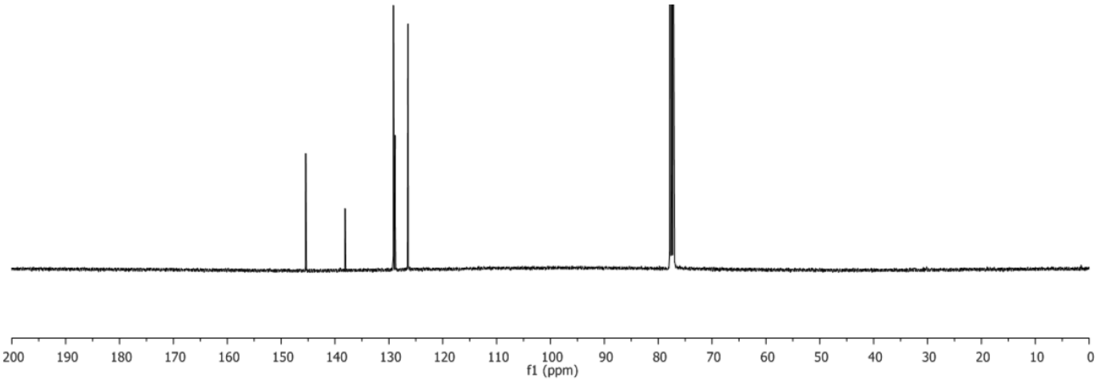
200 190 180 170 160 150 140 130 120 110 100 90 80 70 60 50 40 30 20 10 0
f1 (ppm)

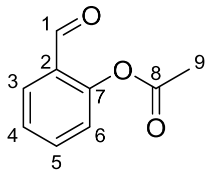
1
2
3
4
5
6
7
8
9
O
O
O

14

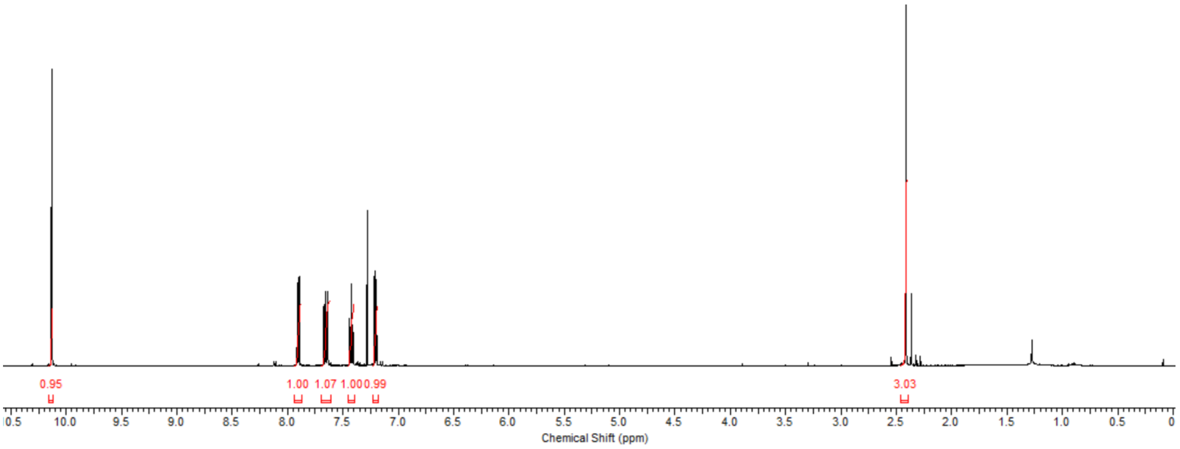
0.95
1.00
1.07
1.00
0.99
3.03
Chemical Shift (ppm)

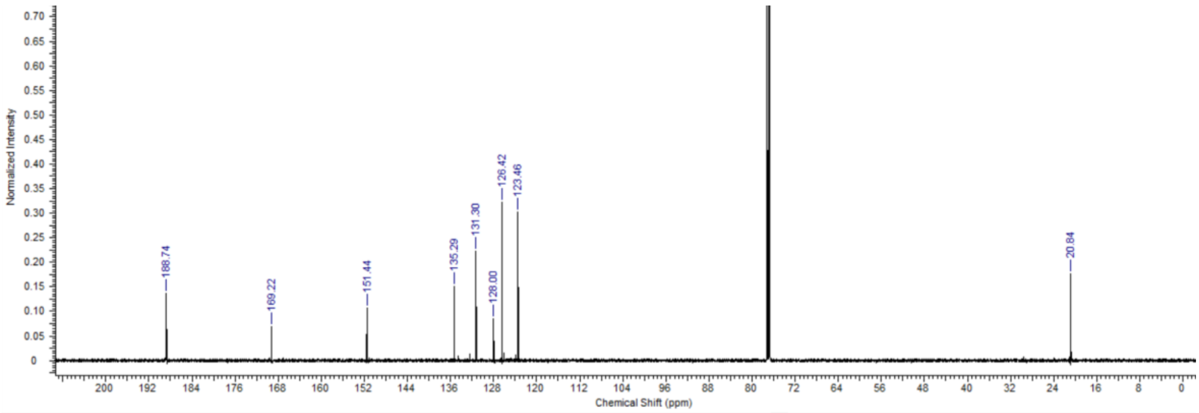
188.74
169.22
151.44
135.29
131.30
128.00
126.42
123.46
20.84
Normalized Intensity
Chemical Shift (ppm)

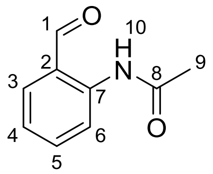
1
O
10
H
N
2
8
9
3
7
4
6
O
5

18

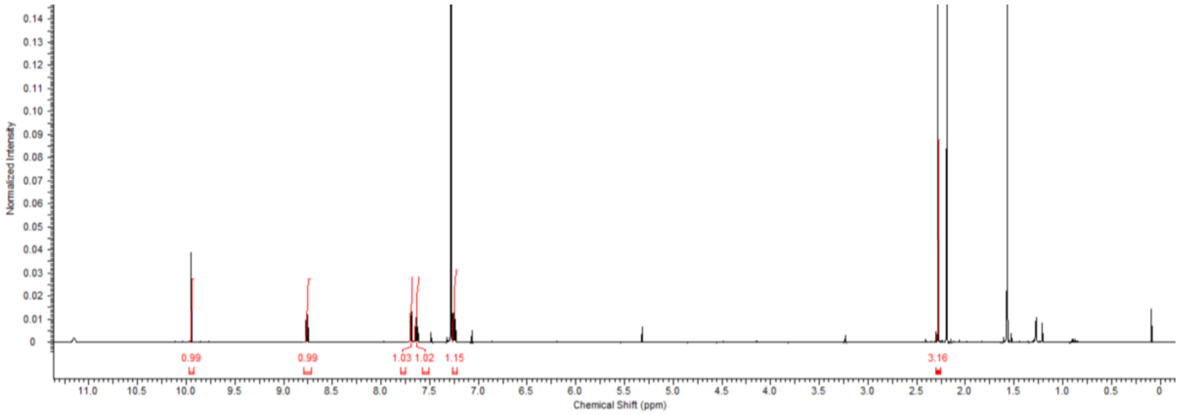
Normalized Intensity
0.99
0.99
1.03
1.02
1.15
3.16
Chemical Shift (ppm)

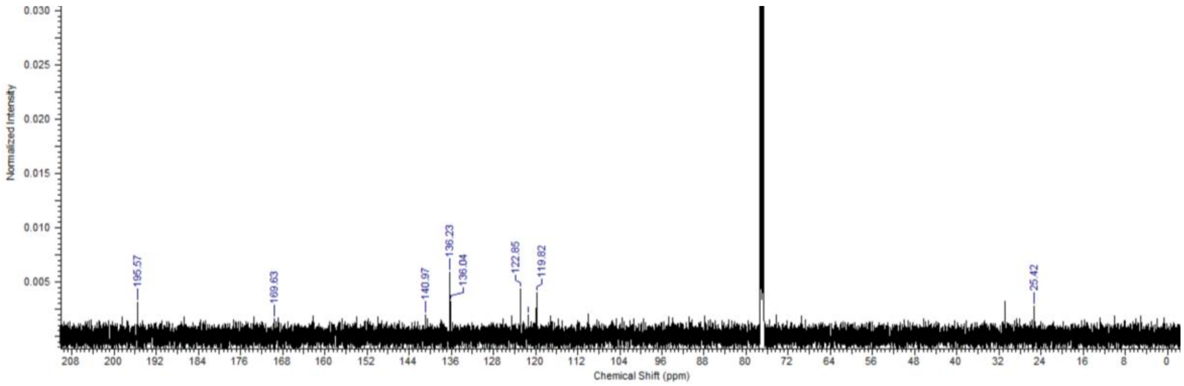
Normalized Intensity
195.57
169.63
140.97
136.23
136.04
122.85
119.82
25.42
Chemical Shift (ppm)

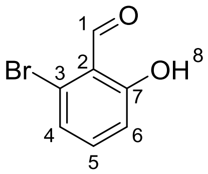
O
1
Br
3
2
OH
8
7
4
6
5

26

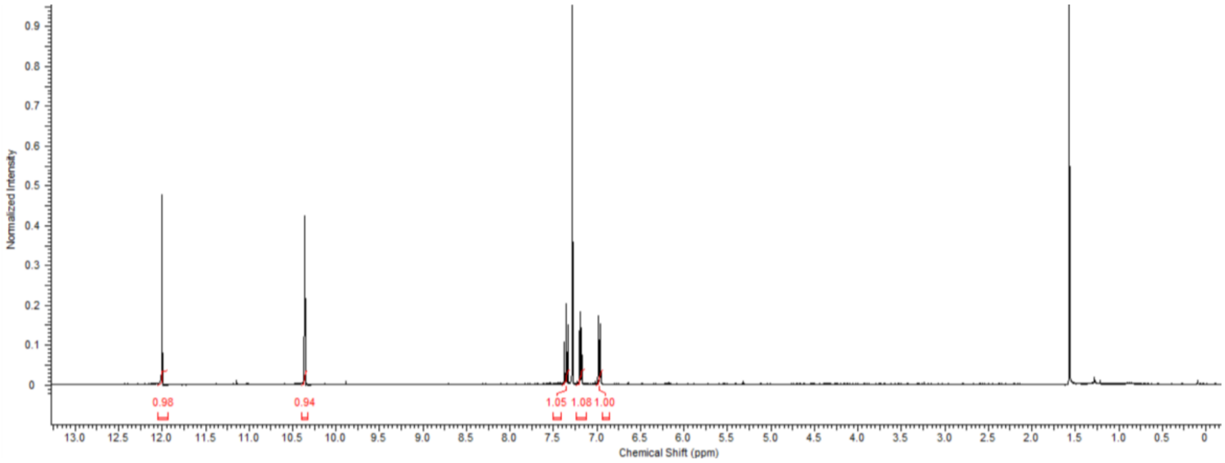
Normalized Intensity
0.98
0.94
1.05
1.08
1.00
Chemical Shift (ppm)

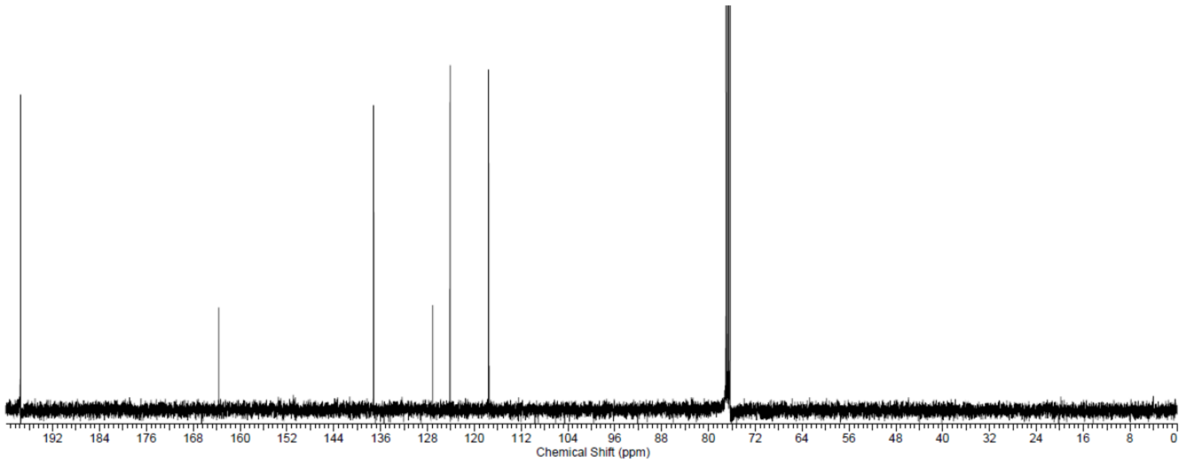
Chemical Shift (ppm)

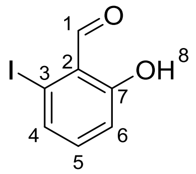

1
O
I
3
2
OH
8
7
4
6
5

28

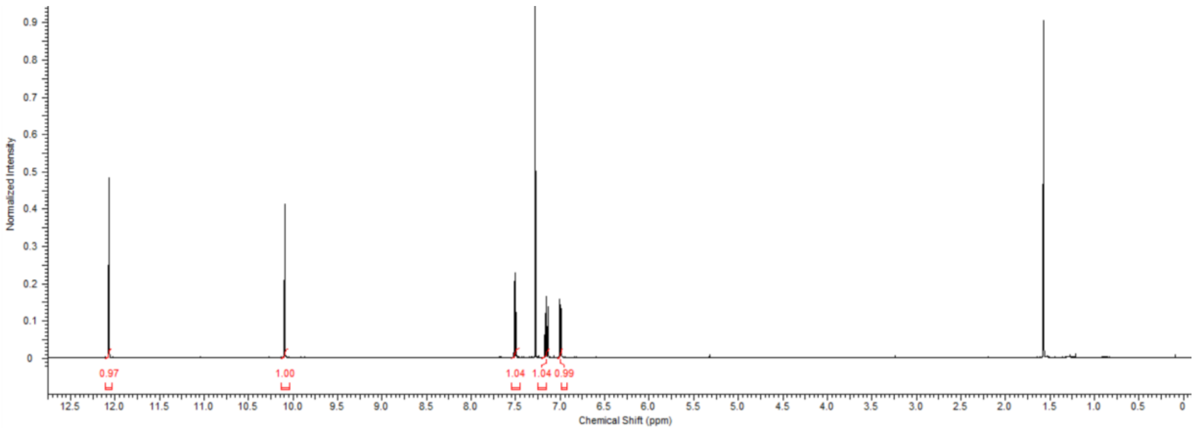

Normalized Intensity
0.97
1.00
1.04
1.04
0.99
Chemical Shift (ppm)

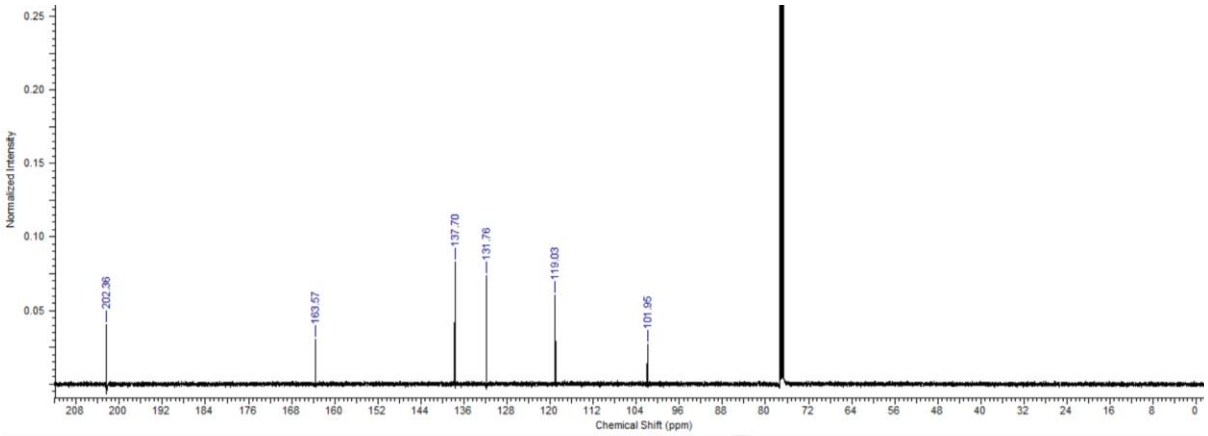

Normalized Intensity
202.36
163.57
137.70
131.76
119.03
101.95
Chemical Shift (ppm)

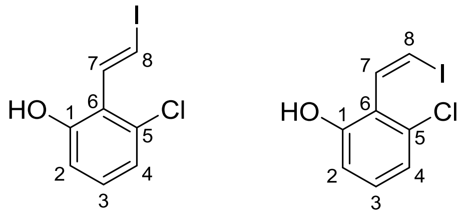

7

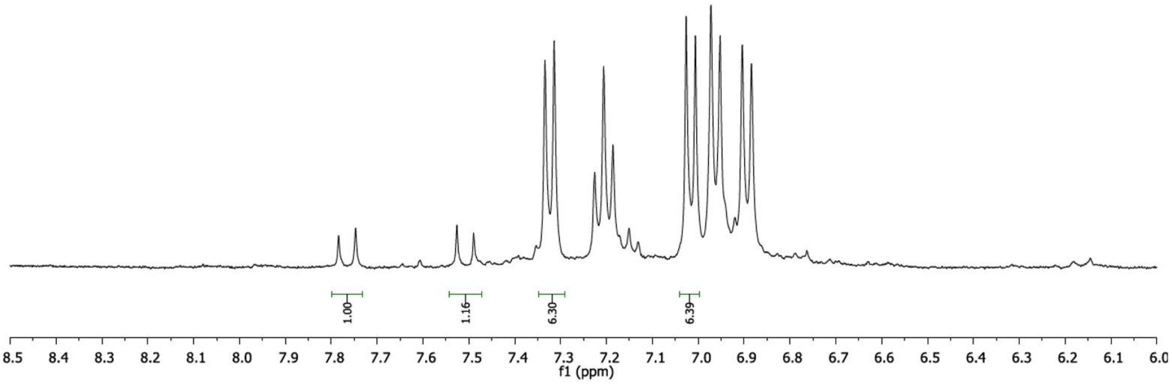

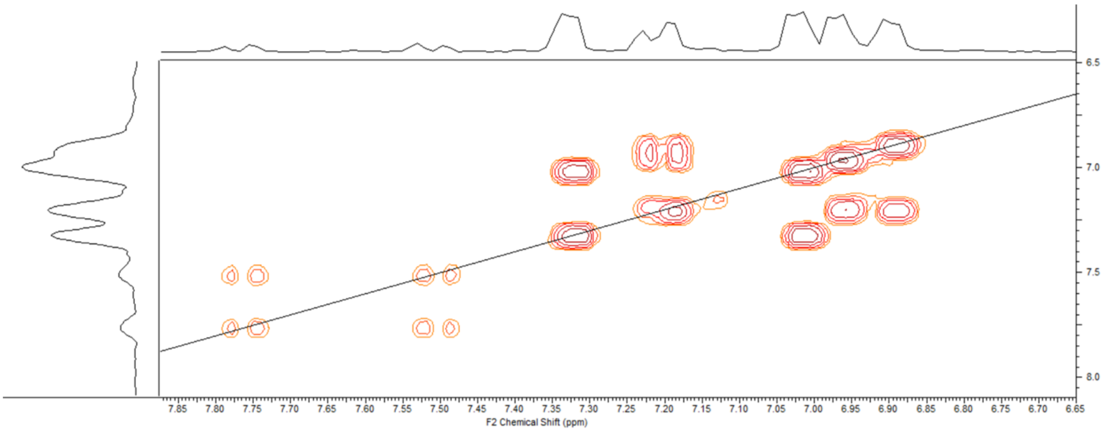

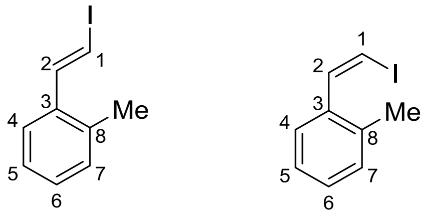

11

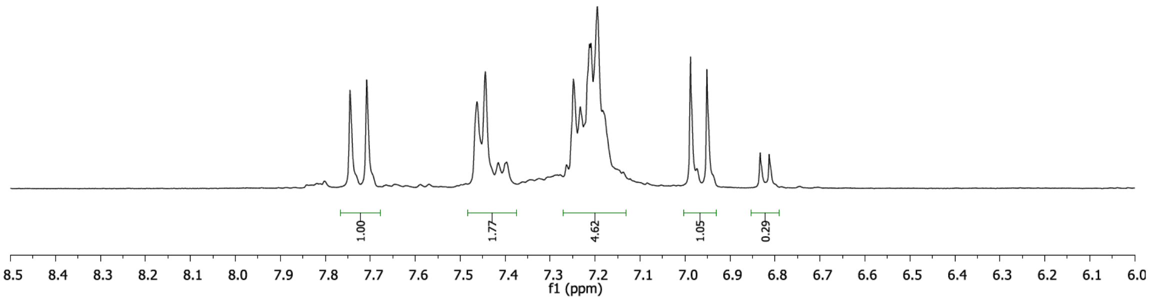

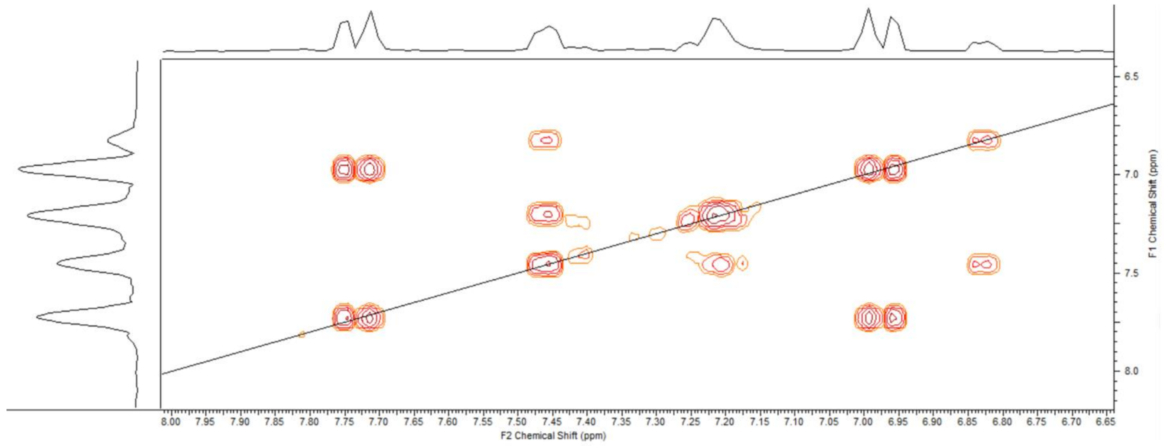

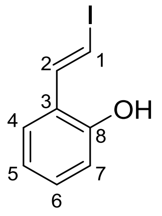

I
2
1
3
OH
4
8
5
7
6

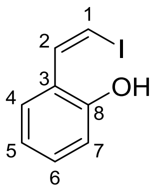

1
2
I
3
OH
4
8
5
7
6

13

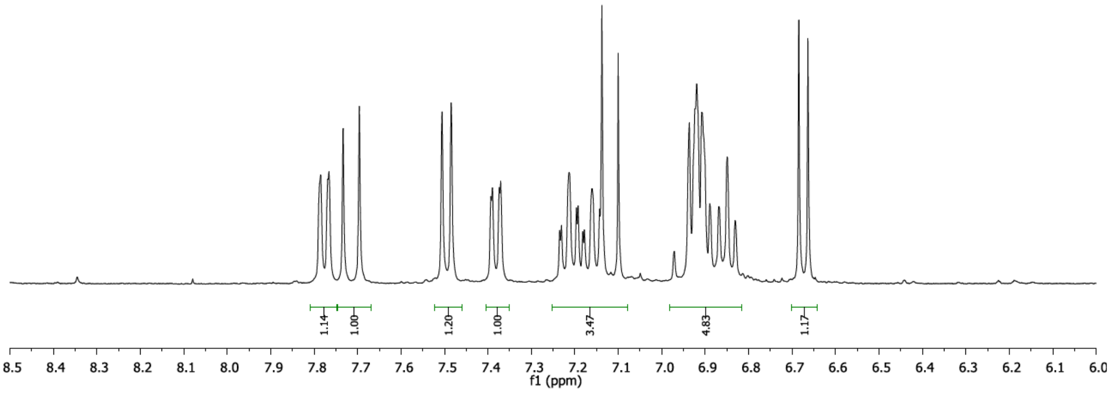

1.14
1.00
1.20
1.00
3.47
4.83
1.17
8.5 8.4 8.3 8.2 8.1 8.0 7.9 7.8 7.7 7.6 7.5 7.4 7.3 7.2 7.1 7.0 6.9 6.8 6.7 6.6 6.5 6.4 6.3 6.2 6.1 6.0
f1 (ppm)

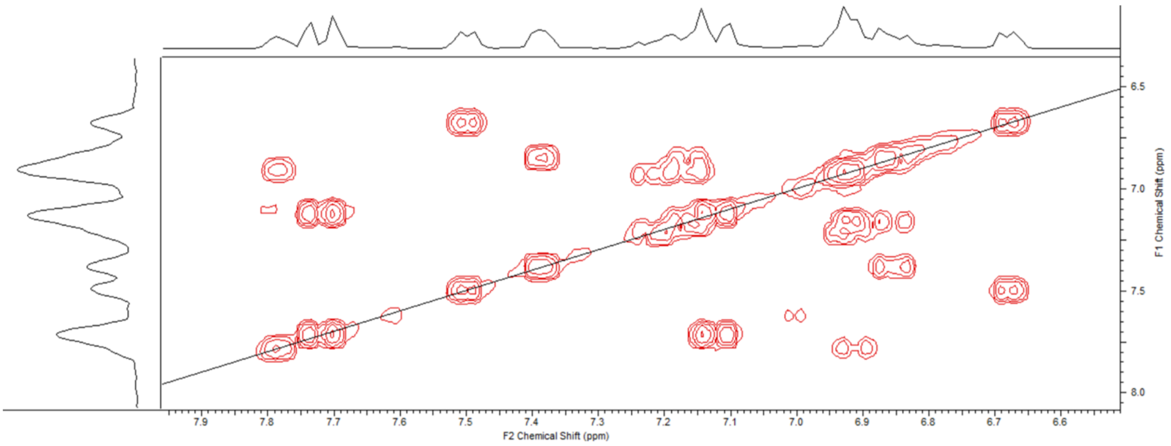

F1 Chemical Shift (ppm)
F2 Chemical Shift (ppm)

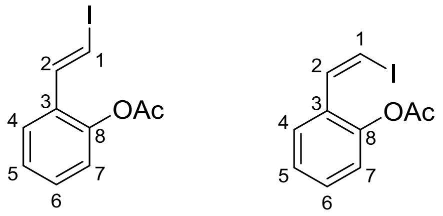

I
OAc
I
OAc

15

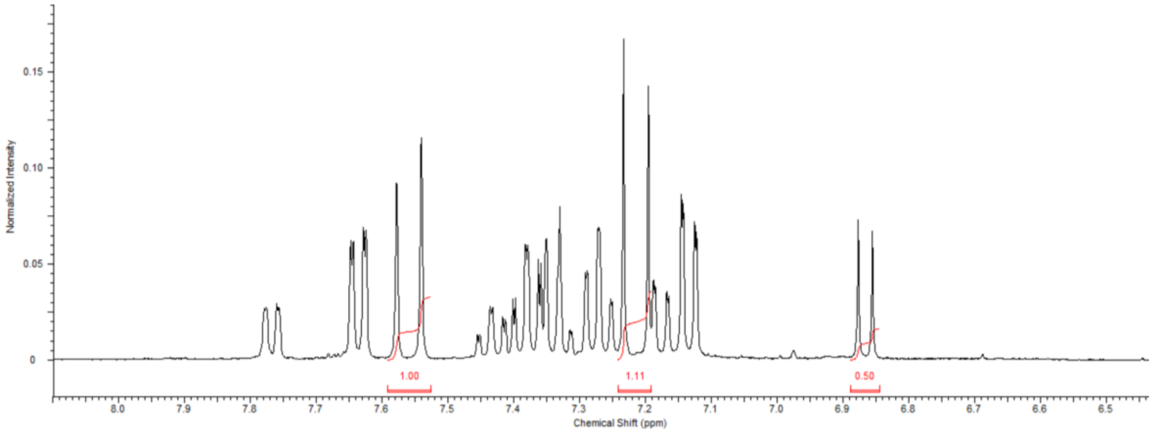

Normalized Intensity
1.00
1.11
0.50
Chemical Shift (ppm)

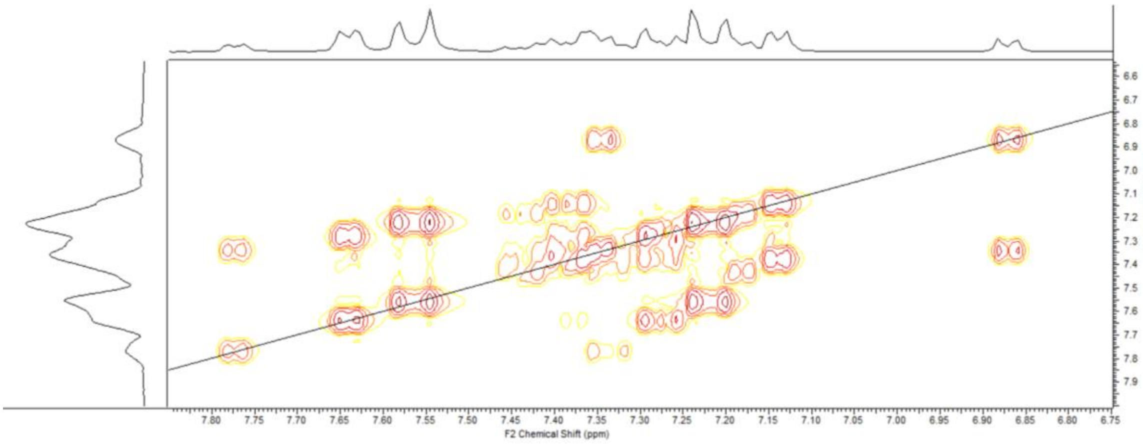

F1 Chemical Shift (ppm)
F2 Chemical Shift (ppm)

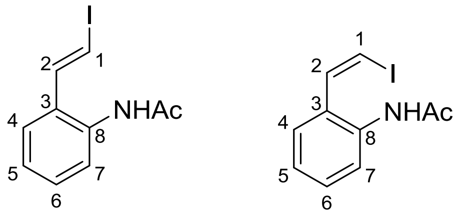

19

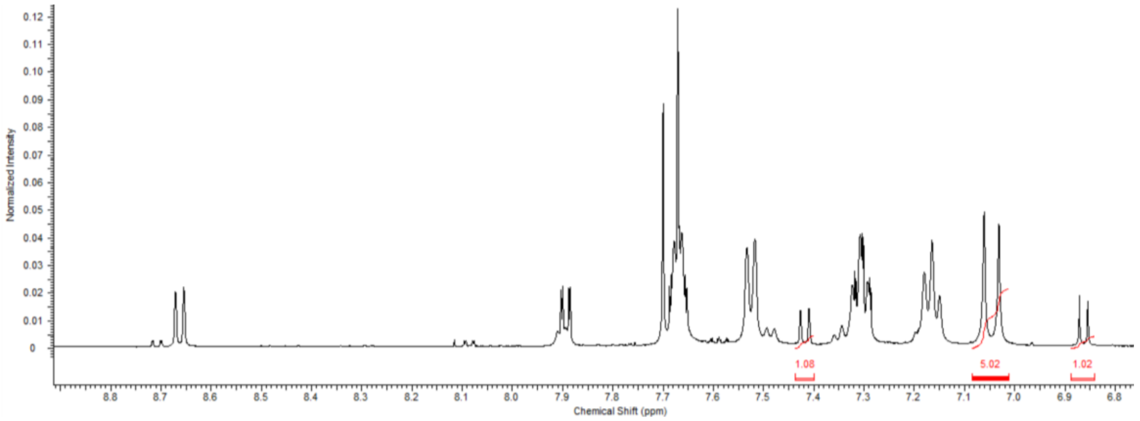

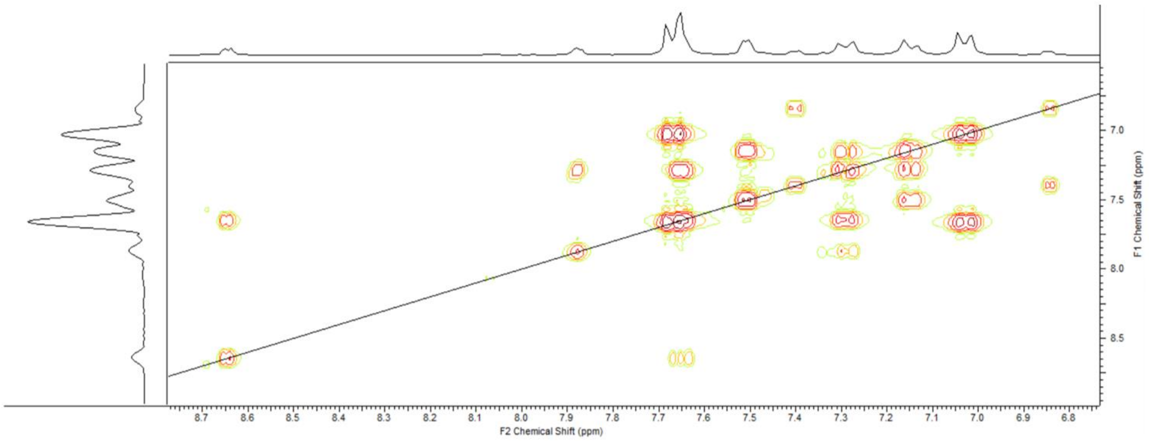

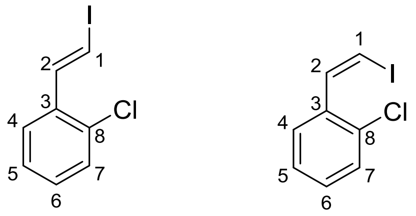
I
Cl
I
Cl

21

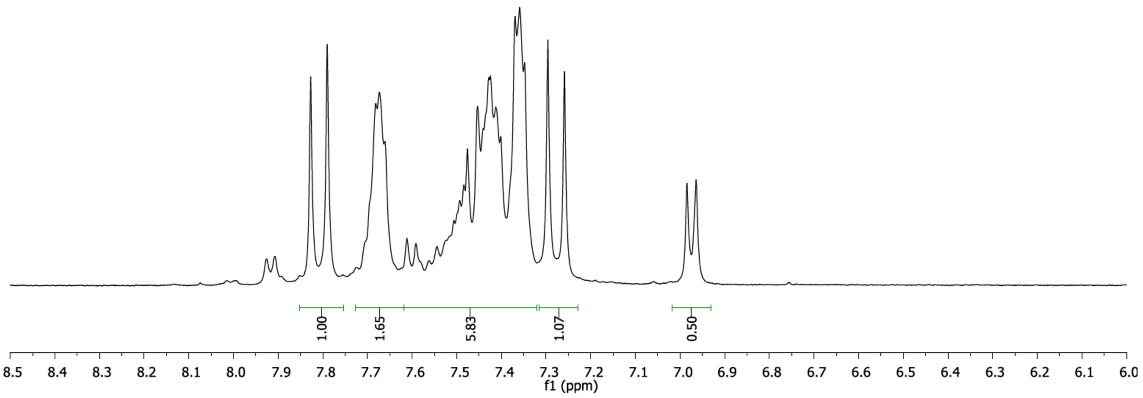
1.00
1.65
5.83
1.07
0.50
f1 (ppm)

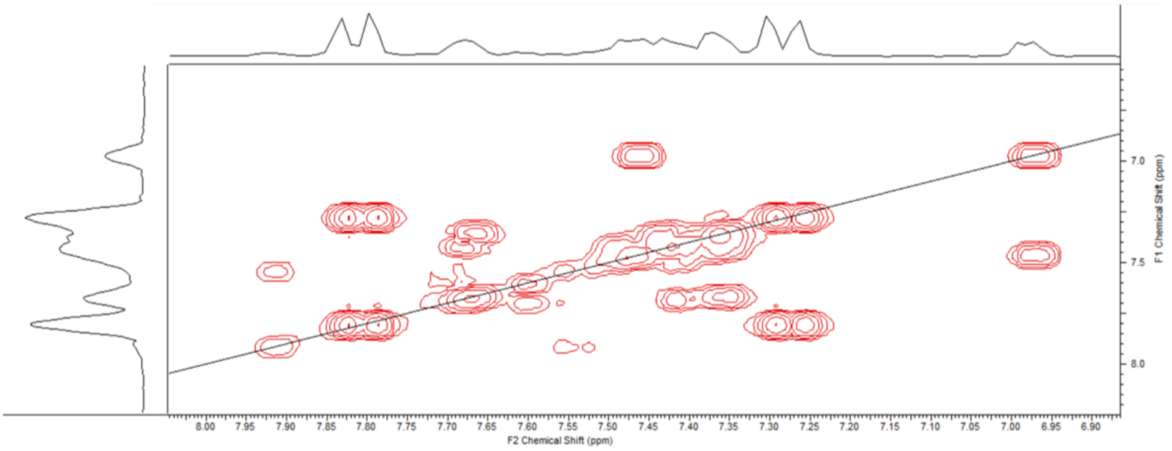
F2 Chemical Shift (ppm)
F1 Chemical Shift (ppm)

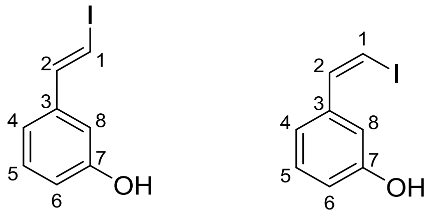
I
2
1
3
4
8
7
5
OH
6
I
2
1
3
4
8
7
5
OH
6

23

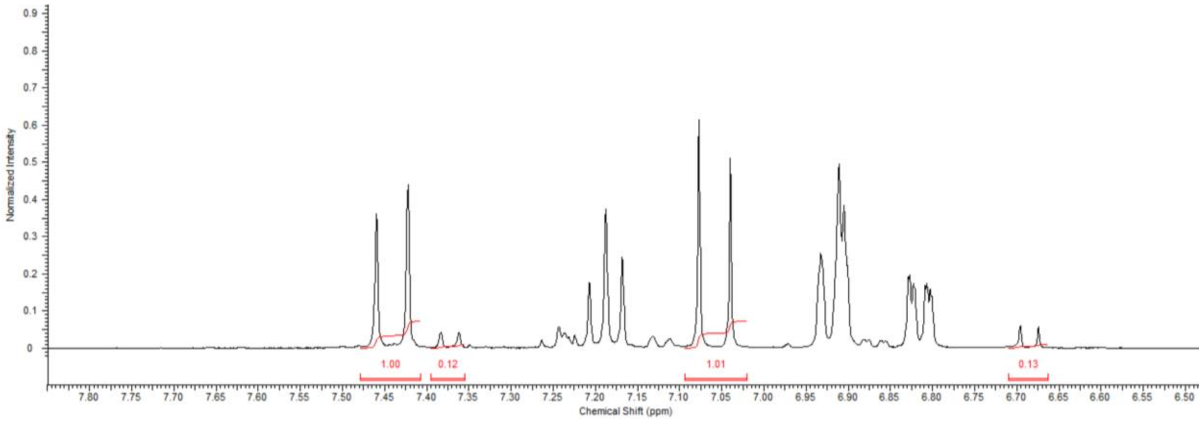
Normalized Intensity
1.00
0.12
1.01
0.13
Chemical Shift (ppm)

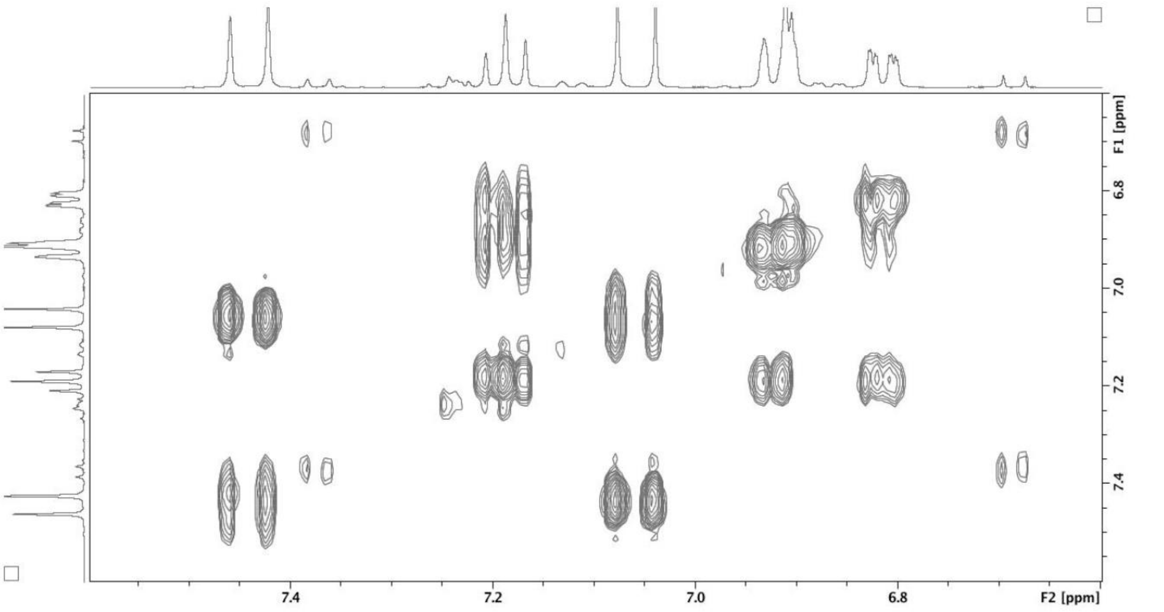
F1 [ppm]
F2 [ppm]

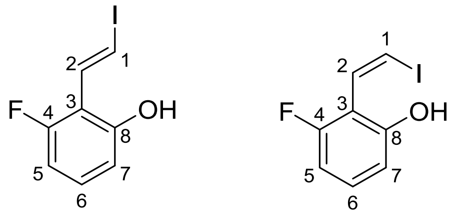
I
F
OH
1
2
3
4
5
6
7
8

25

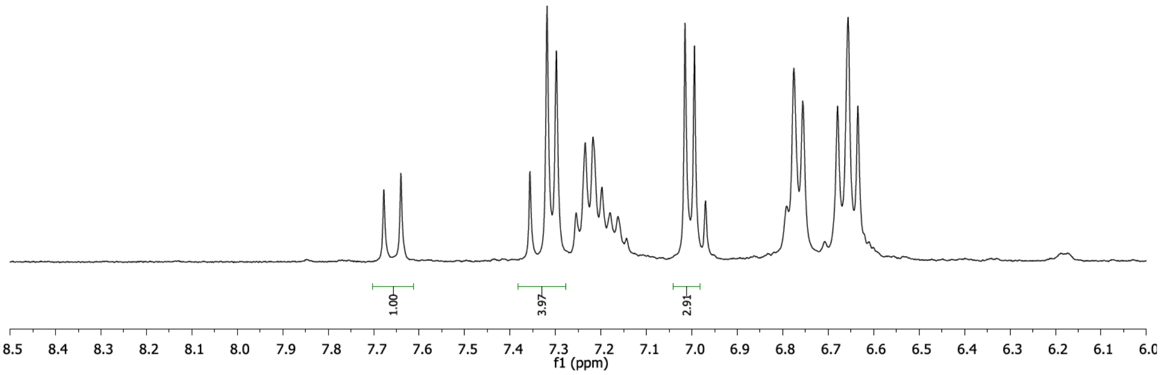
1.00
3.97
2.91
f1 (ppm)

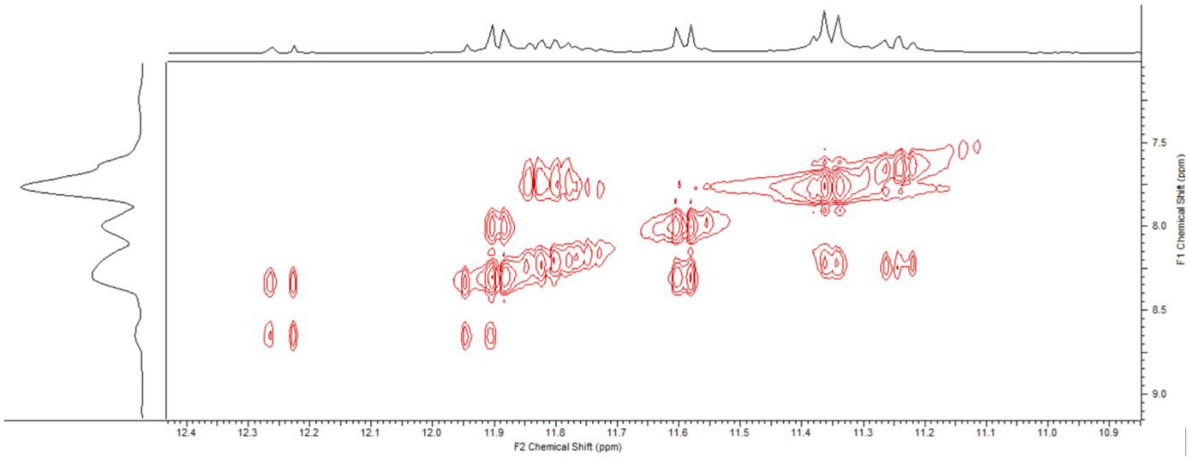
F2 Chemical Shift (ppm)
F1 Chemical Shift (ppm)

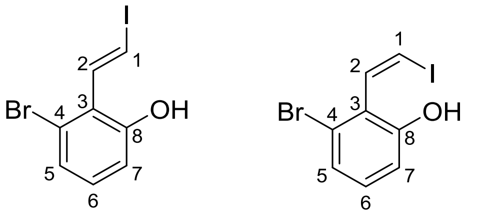
I
Br
OH
1
2
3
4
5
6
7
8

27

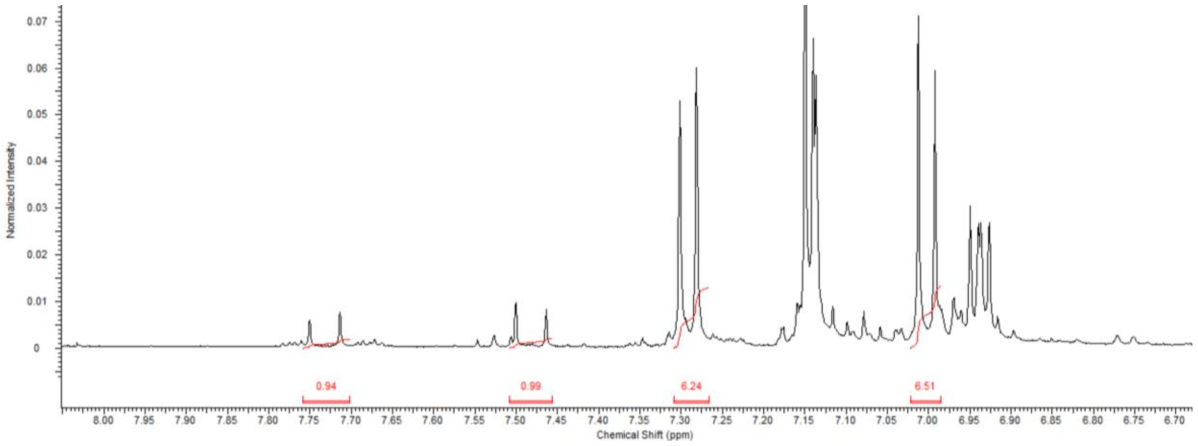
Normalized Intensity
Chemical Shift (ppm)
0.94
0.99
6.24
6.51

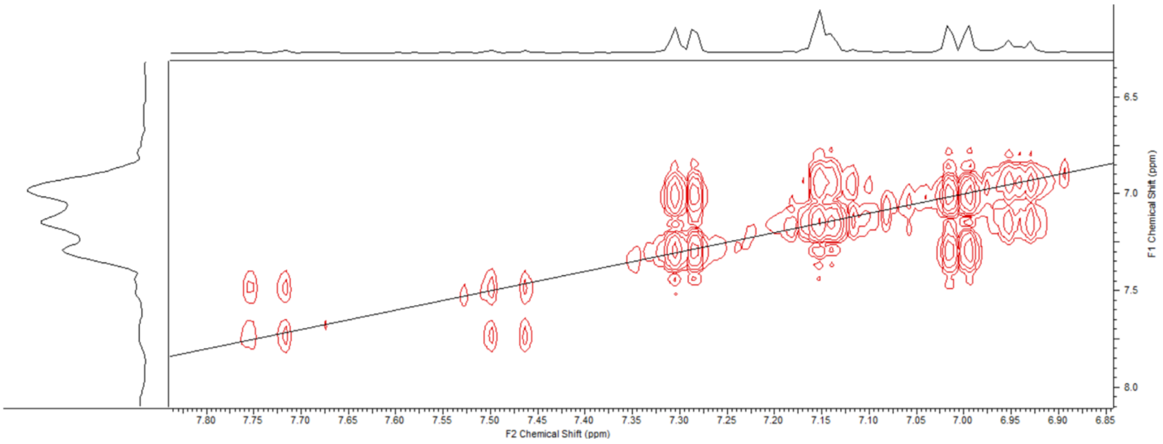
F2 Chemical Shift (ppm)
F1 Chemical Shift (ppm)

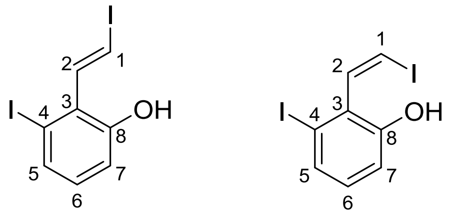
I
2
1
I
4
3
OH
8
5
7
6
1
2
I
I
4
3
OH
8
5
7
6

29

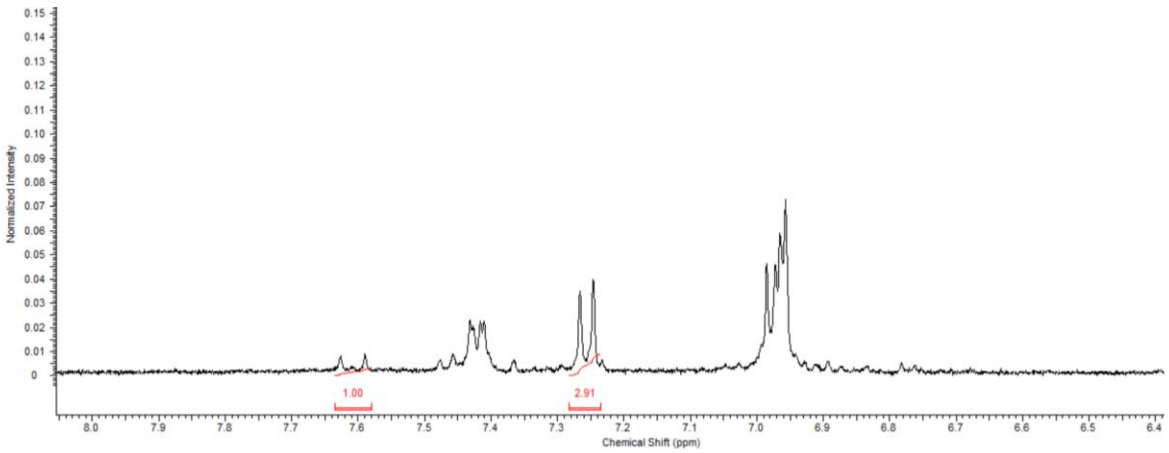
Normalized Intensity
1.00
2.91
Chemical Shift (ppm)

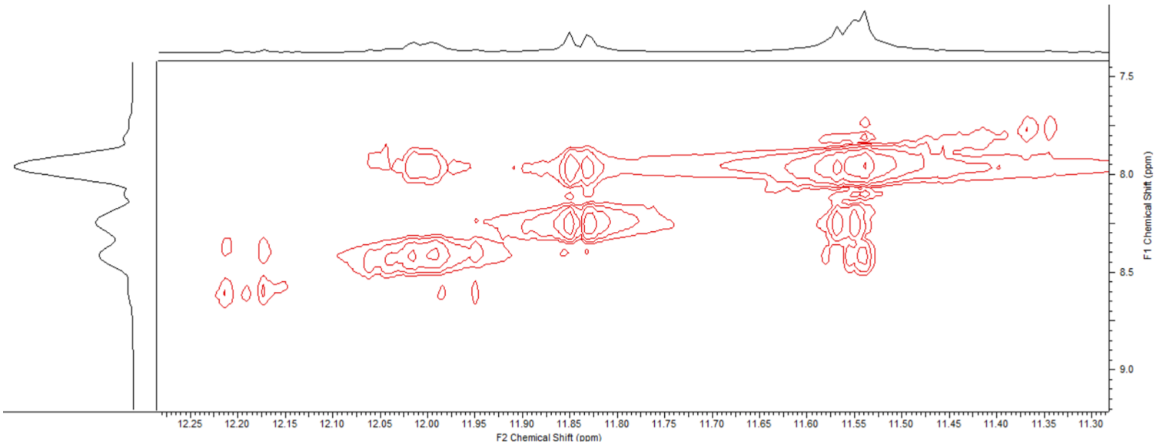
F1 Chemical Shift (ppm)
F2 Chemical Shift (ppm)

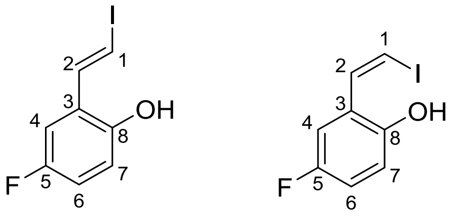

I
OH
F

31

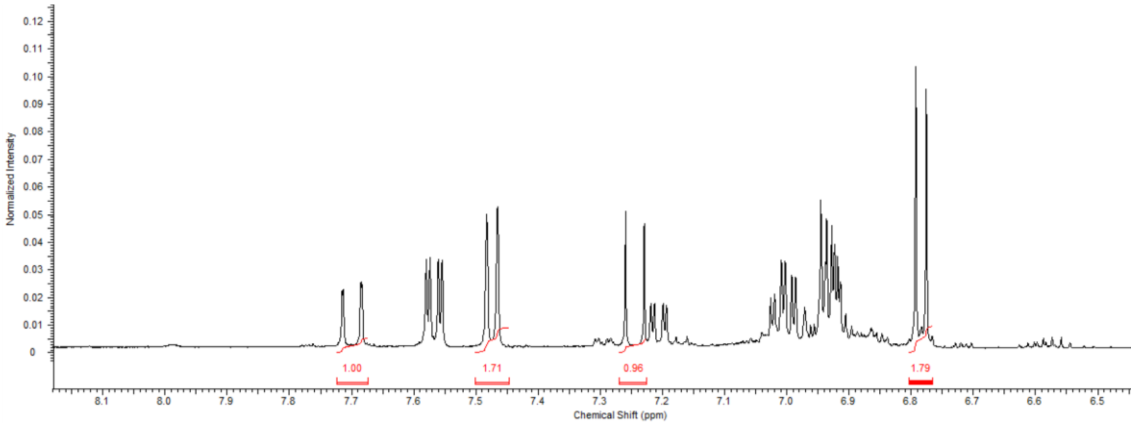

Normalized Intensity
1.00
1.71
0.96
1.79
Chemical Shift (ppm)

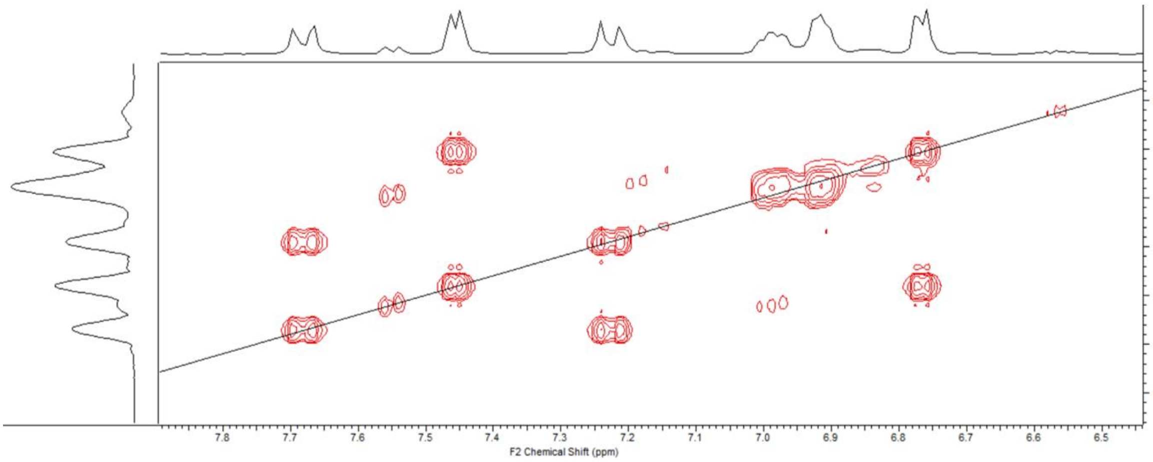

F2 Chemical Shift (ppm)
F1 Chemical Shift (ppm)

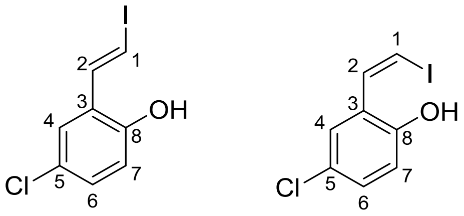
I
2
1
3
OH
4
8
Cl
5
7
6
1
2
I
3
OH
4
8
Cl
5
7
6

33

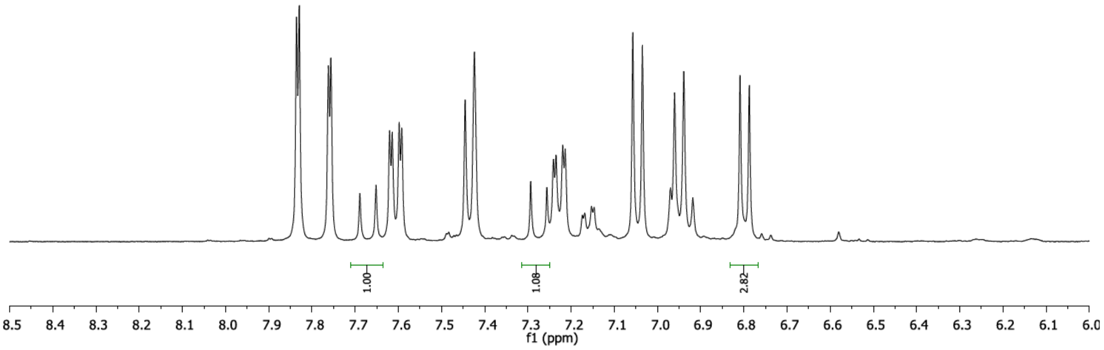
1.00
1.08
2.82
8.5 8.4 8.3 8.2 8.1 8.0 7.9 7.8 7.7 7.6 7.5 7.4 7.3 7.2 7.1 7.0 6.9 6.8 6.7 6.6 6.5 6.4 6.3 6.2 6.1 6.0
f1 (ppm)

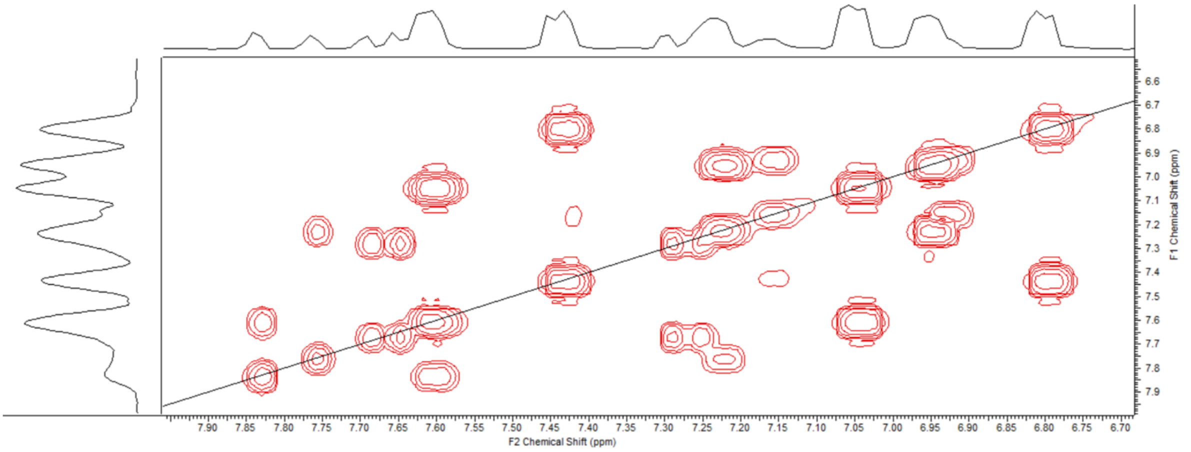
F1 Chemical Shift (ppm)
F2 Chemical Shift (ppm)

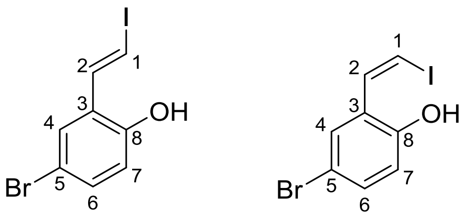

I
OH
Br
I
OH
Br

35

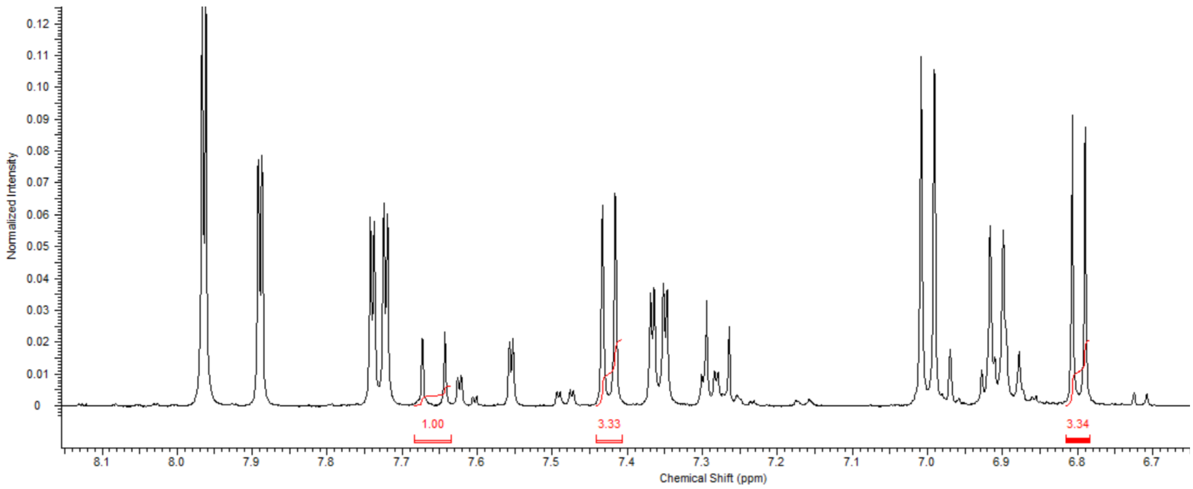

Normalized Intensity
1.00
3.33
3.34
Chemical Shift (ppm)

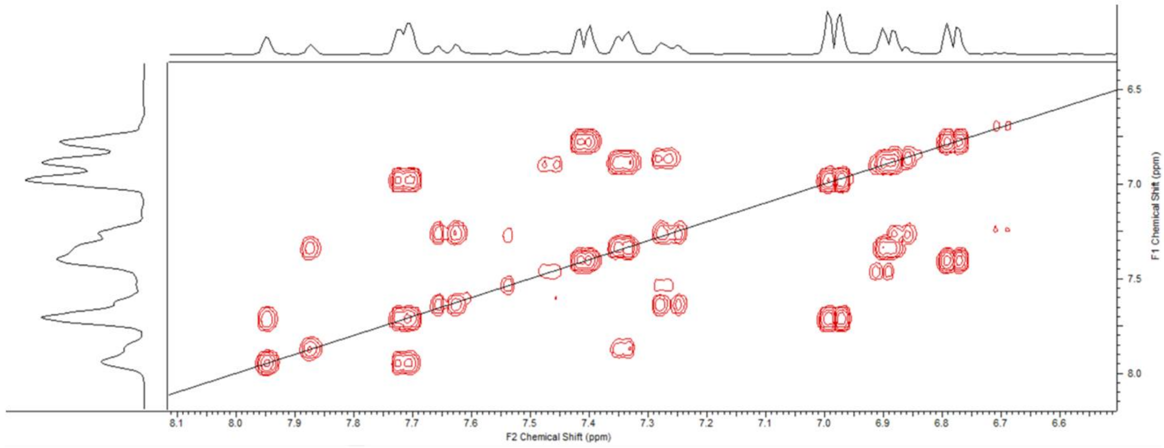

F1 Chemical Shift (ppm)
F2 Chemical Shift (ppm)

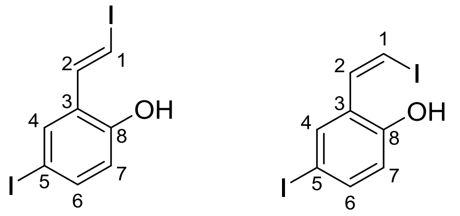

OH
I

37

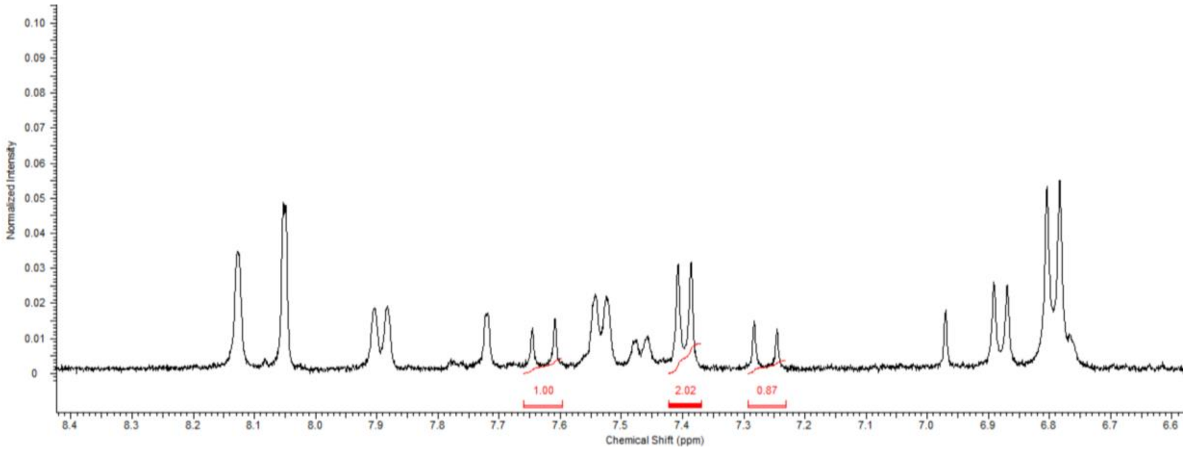

Normalized Intensity
1.00
2.02
0.87
Chemical Shift (ppm)

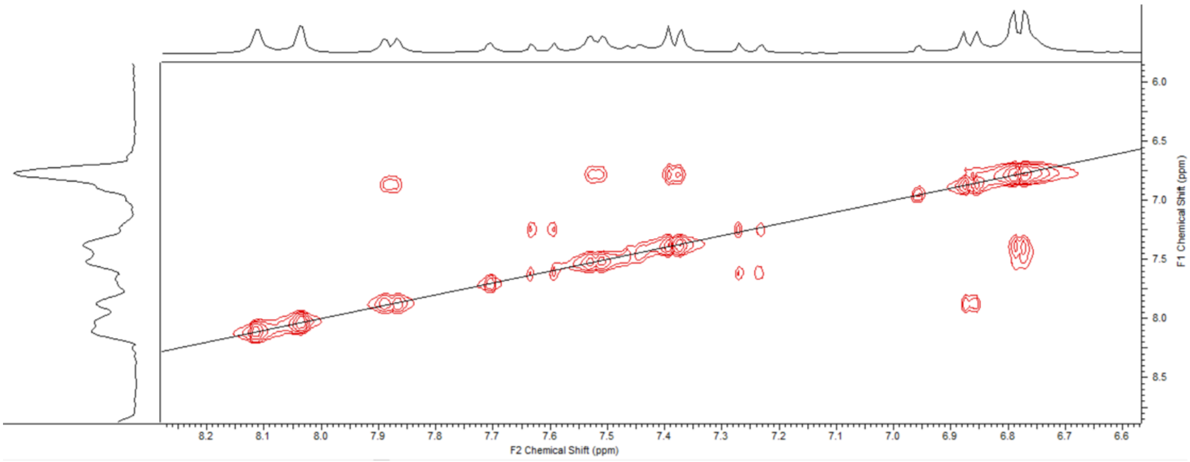

F2 Chemical Shift (ppm)
F1 Chemical Shift (ppm)

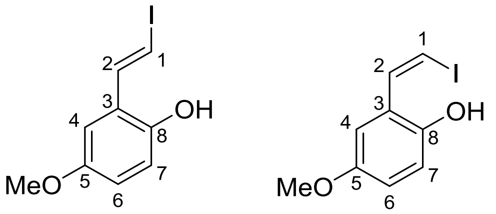

39

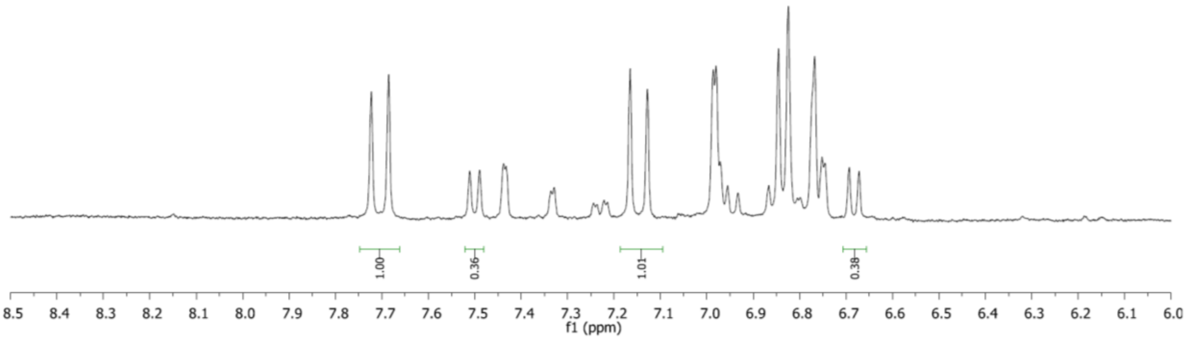

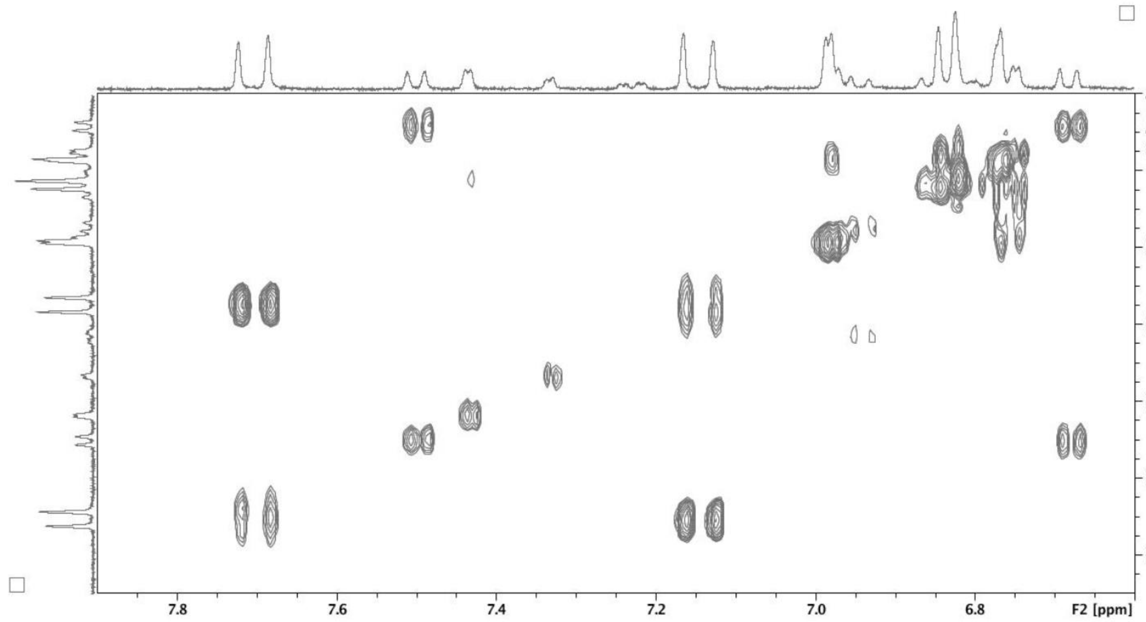

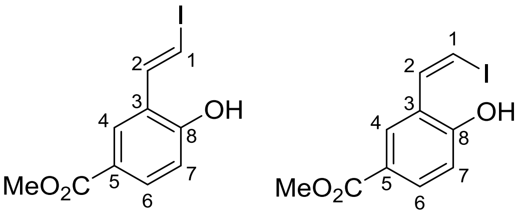
I
1
2
3
4
OH
8
7
6
5
MeO2C
I
1
2
3
4
OH
8
7
6
5
MeO2C

41

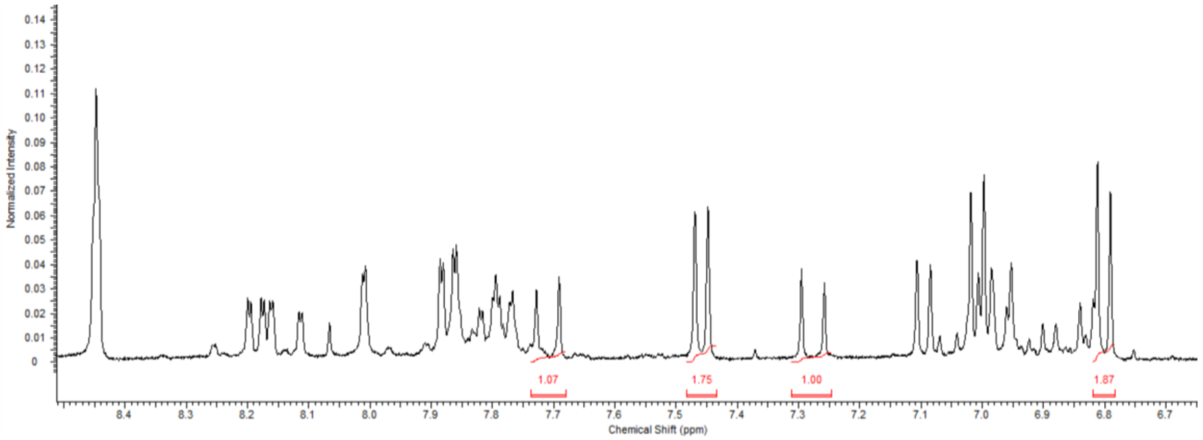
Normalized Intensity
Chemical Shift (ppm)
1.07
1.75
1.00
1.87

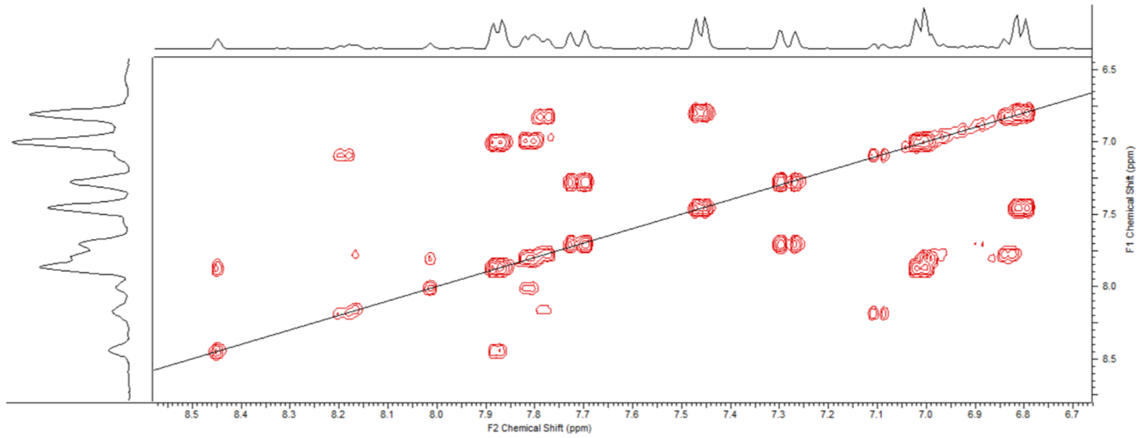
F2 Chemical Shift (ppm)
F1 Chemical Shift (ppm)

Control experiment

Reaction carried out in presence of 0.5 equiv salicylaldehyde.

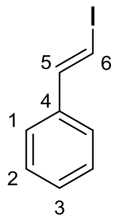

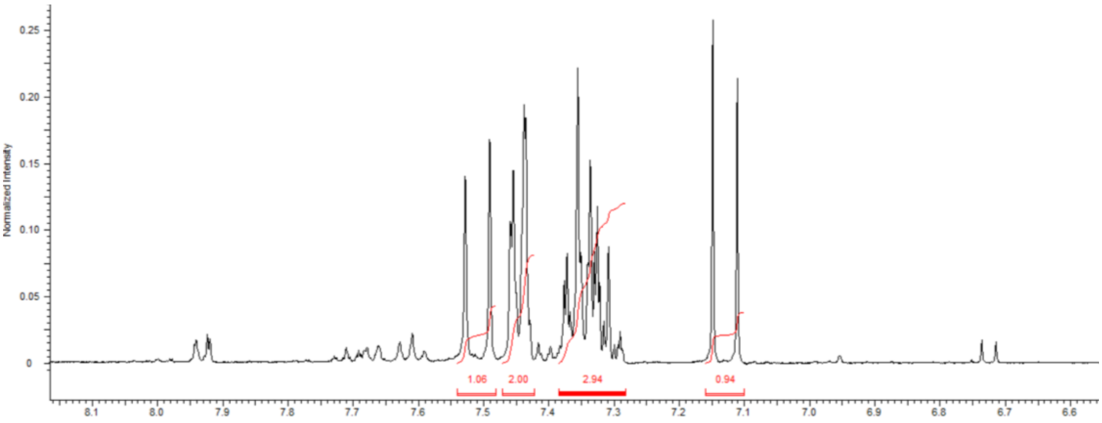

Scale-up experiment

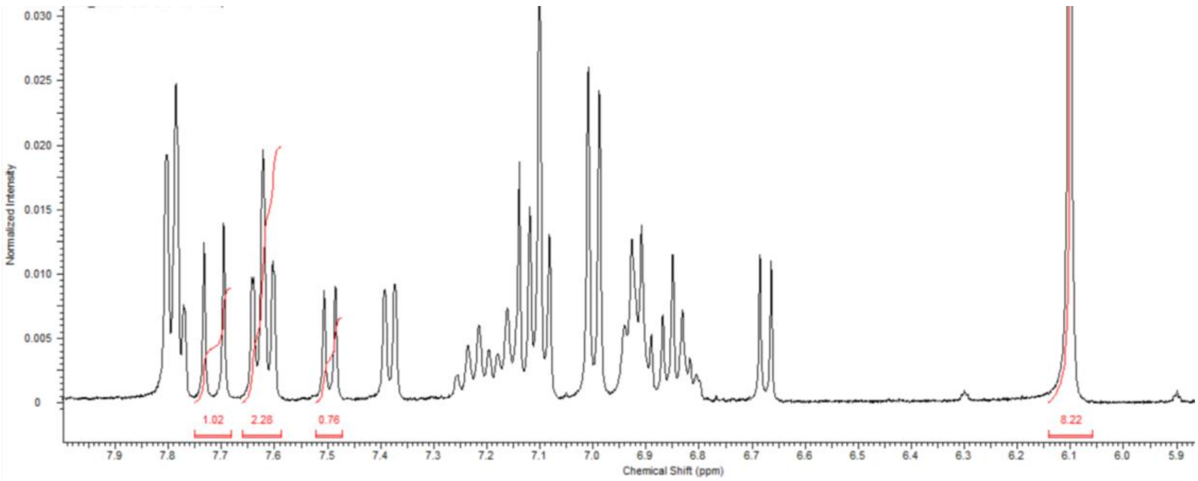

Supplement: File 1 — Experimental procedures and analytical data. [file Beilstein_J_Org_Chem-13-323-s001.pdf]
